# Development of Functional Foods: A Comparative Study on the Polyphenols and Anthocyanins Content in Chokeberry and Blueberry Pomace Extracts and Their Antitumor Properties

**DOI:** 10.3390/foods13162552

**Published:** 2024-08-16

**Authors:** Loredana Stanca, Liviu Bilteanu, Oana Crina Bujor, Violeta Alexandra Ion, Andrei Cătălin Petre, Liliana Bădulescu, Ovidiu Ionut Geicu, Aurelia Magdalena Pisoschi, Andreea Iren Serban, Oana-Mărgărita Ghimpeteanu

**Affiliations:** 1 Faculty of Veterinary Medicine, University of Agronomic Sciences and Veterinary Medicine Bucharest, 105 Blvd, Splaiul Independenței, 050097 Bucharest, Romania; loredana.stanca@fmvb.usamv.ro (L.S.); liviu.bilteanu@fmvb.usamv.ro (L.B.); ovidiu-ionut.geicu@fmvb.usamv.ro (O.I.G.); aurelia-magdalena.pisoschi@fmvb.usamv.ro (A.M.P.); margarita.ghimpeteanu@fmvb.usamv.ro (O.-M.G.); 2 Research Center for Studies of Food Quality and Agricultural Products, University of Agronomic Sciences and Veterinary Medicine of Bucharest, 59 Mărăşti Blvb, 011464 Bucharest, Romania; oana.bujor@qlab.usamv.ro (O.C.B.); violeta.ion@qlab.usamv.ro (V.A.I.); andrei.petre@qlab.usamv.ro (A.C.P.); liliana.badulescu@usamv.ro (L.B.); 3 Faculty of Biology, University of Bucharest, 91-95 Blvd, Splaiul Independenței, 050095 Bucharest, Romania

**Keywords:** chokeberry pomace, blueberry pomace, polyphenol, anthocyanins, health benefits, antitumor, antioxidative, cytokines, Akt, p-Erk

## Abstract

Developing of functional foods is a promising strategy to reduce the increasing burden of colorectal cancer worldwide. Fruit pomace, particularly polyphenol and anthocyanin-rich chokeberry and blueberry, is a valuable ingredient for functional foods and nutraceuticals. Our study aimed to evaluate the anti-inflammatory and antiproliferative effects of chokeberry and blueberry pomace extracts on C2BBe1 colorectal carcinoma cells and explore the underlying signaling pathways. We analyzed both pomace extracts for total polyphenols and anthocyanins using Folin-Ciocalteu method and ultra-performance liquid chromatography, while antioxidative activity was assessed via the 2,2-diphenyl-1-picrylhydrazyl radical scavenging method. We evaluated the in vitro anti-inflammatory and antiproliferative effects using trypan blue exclusion, MTT and LDH assays, and assessed protein levels of p-Erk1/2, Akt-1, STAT1, STAT3, occludin, oxidized proteins, and MDA-protein adducts through western blotting, as well as analysis of a 37-plex panel of inflammatory markers. Chokeberry extracts exhibited higher total polyphenol content, anthocyanin levels, and antioxidative activity compared to blueberry extracts, however, blueberry extracts effects on cell viability and proliferation in C2BBe1 cells were stronger. Both fruit pomaces induced non-inflammatory cell death characterized by membrane integrity loss, beneficial in cancer therapy. Our data suggests chokeberry’s cytotoxicity may be mediated by Erk signaling and Akt-1 inhibition, while blueberry uniquely decreased occludin levels. These berries pomaces’ potential to mitigate cancer risks and enhance treatment efficacy is promising, warranting further investigation for functional foods development.

## 1. Introduction

Colorectal cancer (CRC) is the third most common cancer globally [1], with over 1.9 million new cases and 930,000 deaths estimated in 2020. By 2040, CRC is projected to increase to 3.2 million new cases and 1.6 million deaths annually, primarily in regions like Eastern Europe [2]. Although environmental and genetic factors are significant in the pathogenesis of colon cancer, extensive research has indicated that nutrition can have both a causal and protective impact on its development [3,4]. CRC is largely preventable through lifestyle changes and early detection, thus developing functional foods and encouraging dietary changes are vital strategies to reduce the prevalence and burden of CRC worldwide.

Approximately 45% of fruits and vegetables are wasted worldwide, which is one of the categories with the highest wastage rate [5]. Nowadays, the food industry is focused on developing functional food formulations and nutraceuticals using by-products from the agro-industry, including fruit pomace and leaves of certain crops, a goal aligned with the zero-waste initiative set forth by the European Union that supports the United Nations Sustainable Development Goal [5,6]. Pomace from red fruits from families such as *Rosaceae* (chokeberry, strawberry, raspberry, blackberry, and sweet cherry) and *Ericaceae* (blueberry, cranberry) have received special attention, due to their high content of polyphenols, vitamins, and dietary fiber with a low content in calories, as well as their health-promoting properties [7]. Being an excellent source of natural antioxidants, the chokeberry (*Aronia melanocarpa* L.) pomace polyphenol content ranges from 31 to 63 mg/g dry weight (dw) [8,9] and in the case of blueberries (*Vaccinium* sp.) it is generally lower and depends in both cases on extraction methods [10,11].

Polyphenols are represented by flavonoids and phenolic acids, with properties dependent on the variation in number and arrangement of the hydroxyl groups, and their alkylation or glycosylation [12]. Flavonoids are a class of plant secondary metabolites characterized by their 15-carbon backbone, which consists of two phenyl rings (A and B) and a heterocyclic ring (C) [13]. Flavonoids consist of flavanols (such as the catechins), anthocyanins, and flavonols (e.g., quercetin and kaempferol), whereas phenolic acids are primarily made up of chlorogenic acid and its isomer, neochlorogenic acid [9]. Flavonols have a 3-hydroxy-pyran-4-one group on the C ring, while flavanols lack this 4-one structure and have a saturated C2-C3 bond. Flavones lack a hydroxyl group at the 3-position but have a double bond between C2 and C3, similar to flavonols. Flavanones also have a saturated C2-C3 bond, like flavanols. Isoflavones have the B ring attached at the 3-position on the C ring, unlike the 2-position attachment in other flavonoids. Among flavonoids, an interesting class of compounds is represented by anthocyanins, characterized by the presence of an oxonium ion on the C ring [12,14] Anthocyanins, produced via the phenylpropanoid pathway, are glucosides of the flavonoid derivatives known as anthocyanidins [15]. 

The antioxidant and anti-radical activities of poliphenols are closely linked to the number and positioning of hydroxyl groups on the aromatic ring. Specifically, hydroxyl groups in the *ortho*-position are key contributors to their powerful antioxidant and radical scavenging abilities, being able to quench reactive radical species by single electron transfer reaction and through hydrogen atom abstraction from phenolic groups [15]. Polyphenols interact with nonpolar compounds in the inner hydrophobic layer of the plasma membrane, reducing the oxidation rate of lipids and proteins. Flavonoids located in the hydrophobic core of membranes obstruct oxidative species, thereby protecting the membrane’s structure and functionality [16,17]. Anthocyanins are colored phenolic compounds commonly found in red and blue fruits, and their content is influenced by factors such as cultivar or variety, growing area, climate, farming methods, harvest time, ripening, seasonal variability, processing and storage conditions, temperature, and light exposure. Berries like blueberries, blackberries, strawberries, raspberries, elderberries and chokeberries are rich in anthocyanins, with levels ranging from about 1 to 18 mg/g dw product [9,10,15,18]. Chokeberries have the highest anthocyanin content, primarily composed of cyanidin glycoside (98.4%), followed by small amounts of malvidin glycoside, pelargonidin glycoside, delphinidin glycoside and petunidin glycoside. Blueberries primarily contain malvidin glycoside and delphinidin glycoside, followed by petunidin glycoside, peonidin glycoside, and cyanidin glycoside [19]. Besides the use as food colorants, these compounds are potentially useful in developing functional food formulations and as nutraceutical ingredients, as they provide numerous beneficial health effects. Many in vitro an in vivo studies have evaluated the biological and pharmacological potential of polyphenols and demonstrated that they possess the capacity to counteract oxidative stress, to act as antimicrobial substances, and to counteract the onset and the progression of diseases such as neurodegenerative, cardiovascular, metabolic diseases and cancer [7,15,20,21]. Anthocyanins, are known for their anticancer and antiangiogenic properties, including in colorectal cancer [20]. For instance, chokeberry extracts have demonstrated a consistent antiproliferative effects in human HT-29 colon cancer cells and modulated tumor suppression genes [22]. Similarly, anthocyanin-rich extracts from bilberry, chokeberry, and grape reduced aberrant crypt foci in rats, correlating with decreased cell proliferation and COX-2 gene expression [23]. Additionally, dietary anthocyanin-enriched purple-fleshed sweet potato significantly suppressed aberrant crypt foci formation in colon mucosal epithelial cells [24], indicating its protective effect against colorectal cancer through apoptotic and anti-proliferative mechanisms. Thus, the development of functional foods with antitumoral properties would represent an alternative in the fight against colorectal cancer, with chokeberry or blueberries pomace being valuable sources of bioactive compounds. 

The aim of this study was to provide analytical data regarding composition in polyphenols and anthocyanins content of chokeberry and blueberry pomace extracts as well as their anti-inflammatory and antiproliferative effects on an adenocarcinoma cell line and the evaluation of possible signaling pathways involved in mediating their effects.

## 2. Materials and Methods

### 2.1. Chemicals and Reagents

The anthocyanin standards delphinidin-3-O-galactoside, cyanidin-3-O-galactoside, cyanidin-3-O-glucoside (kuromanin chloride), cyanidin-3-O-arabinoside and cyanidin-3-O-xyloside were purchased from Extrasynthese (Genay, France). HPLC gradient grade acetonitrile and methanol were purchased from Honeywell Riedel-de Haën (Seelze, Germany), 37% hydrochloric acid from Merck (Darmstadt, Germany) and formic acid from Honeywell Fluka (Steinheim, Germany). In addition, gallic acid was purchased from Roth GmbH, methanol, Folin & Ciocalteu’s phenol reagent 2 N and DPPH (1,1-diphenyl-2-picrylhydrazyl) radical were from Sigma-Aldrich Chemie GmbH (Riedstrasse, Steinheim). Trolox (6-hydroxy-2,5,7,8-tetramethylchroman-2-carboxylic acid) was purchased from Acros Organics, Fisher Scientific (Geel, Belgium). 

### 2.2. Plant Material and Pomace Preparation

Fully ripened chokeberry (*Aronia melanocarpa*) and blueberry (*Vaccinium corymbosum*) fruits were collected from a farm situated in Crovu (44°35′23.77″ N 25°32′13.6″ E) at 65 km from Bucharest, Romania in 2022. The fruits were squeezed and the pomaces were frozen in an ultra-low temperature freezer (MDF-594-PE, Panasonic Corporation, Osaka, Japan) at −80 °C for 24 h. After freezing, the samples were lyophilized in a freeze dryer (Alpha 2-4 LSCplus, Martin Christ Gefriertrocknungsanlagen GmbH, Osterode am Harz, Germany) at a pressure of 0.5 mPa and with the shelf temperature of −55 °C for 45 h [25]. The lyophilized pomaces were then ground into a fine powder using a Grindomix GM 200 mill (Retsch, Haan, Germany). Samples were kept in cool, dry and dark storage conditions until further processing. The solvents used for pomace extracts preparation were ethanol (100% *v*/*v*), ethanol-distilled water (70:30 and 50:50% *v*/*v*), water with a vinegar content of 0.5% and distilled water. The dry weight pomace to solvent ratio was 1:10 (*w*/*v*). The extraction was performed for 15 min in the ultrasonic bath (Sonorex Super RK 102H, Bandelin Electronic GmbH & Co. KG, Berlin, Germany), at 35 kHz and 37 °C then centrifuged for 5 min at 5000× *g* and 4 °C. After centrifugation, the supernatants were filtered through cellulose or Teflon filters (depending on the type of solvent). The extraction and all tests were carried out in triplicate. Before the UPLC measurement, the supernatant was filtered through a 0.45 µm membrane filter.

### 2.3. Total Phenolic Content (TPC)

The total phenolic content of the extract solutions was determined by the Folin—Ciocalteu spectrophotometric method [25]. A 2.5 mL volume of aqueous Folin—Ciocalteu reagent (1:10 dilution factor) was added to 0.5 mL of extract. The mixture was incubated for 2 min at room temperature, and after incubation, 2 mL of sodium carbonate (7.5%) was added. The mixture was heated in a water bath at 50 °C for 15 min and finally cooled in a water-ice bath. A mixture of solvent and reagents was used as a blank. Absorbance was measured at 760 nm using a spectrophotometer (Specord 210 Plus UV-Vis, Analytik Jena, Jena, Germany). Total phenolic content was expressed as mg Gallic acid equivalents per g dry weight (mg GAE/g dw). Triplicates of independent extract solutions were analyzed.

### 2.4. Determination of Antioxidant Activity Using the 2,2-Diphenyl-1-picrylhydrazyl (DPPH) Radical Scavenging Method

The DPPH radical’s stable structure, featuring a nitrogen atom bonded to two phenyl rings and a picrylhydrazyl moiety, makes it an ideal probe for antioxidant assays due to the delocalization of its unpaired electron. The DPPH assay evaluates antioxidant activity by mixing DPPH radicals with a test sample. Antioxidants in the sample reduce DPPH to a non-radical form, causing a color change from deep purple to yellow, which is quantitatively measured. DPPH radical scavenging activity of extracts was determined by a slightly modified method developed by Bujor et al. [26]. A volume of 200 µL extract was added to 2 mL of 0.2 mM solution of DPPH radical in methanol. The mixture was incubated in dark under continuous homogenization at 500 rpm, for 30 min, using a homogenizer (KS 260, IKA, Staufen, Germany). The decrease in absorbance at 517 nm was measured using a spectrophotometer (HP 8453 Diode Array G1103A, Agilent Technologies, Santa Clara, CA, USA). The calibration curve was prepared using 6-hydroxy-2,5,7,8-tetramethylchroman-2-carboxylic acid (Trolox). The antioxidant activity of each extract was expressed as milligram of Trolox equivalents (TE) per gram of dry weight pomace (mg TE/g dw). 

### 2.5. Identification and Quantification of Anthocyanins by Ultra Performance Liquid Chromatography (UPLC) Analysis

The purification and anthocyanins UPLC analysis was carried out according to a procedure described by Bujor et al. with some modifications [26]. Before UPLC analysis, preliminary purification of anthocyanins from the pomace extracts was performed using solid-phase extraction (SPE). Firstly, 5 mL of ethanol extracts were concentrated under nitrogen in order to evaporate the ethanol. The remaining aqueous extract was made up to 5 mL with 0.3% HCl aqueous solution and purified by a Strata C18-E cartridge (55 μm, 70 Å, 200 mg/mL, Phenomenex, Torrance, CA, USA). The aqueous extracts were not concentrated under nitrogen. The cartridge was conditioned with two column volumes of 0.01% HCl in methanol followed by two volumes of 0.01% aqueous HCl (*v*/*v*) to remove remaining methanol. Afterwards, the extracts were loaded onto the mini-column and washed with one volume of 0.01% aqueous HCl to remove compounds not adsorbed. The anthocyanins were eluted with 0.01% HCl in methanol and immediately analyzed by UPLC.

A Waters ACQUITY UPLC System (Waters, Milford, MA, USA) coupled to UV/VIS diode-array detector (UV/VIS PDA) was used. Separation was performed on a reverse-phase Eclipse Plus C18 column (100 mm × 2.1 mm i.d., 1.8 µm; Agilent Technologies) at 30 °C. A binary solvent system with solvent A (1% formic acid in water, *v*/*v*) and solvent B (1% formic acid in acetonitrile) and the following elution gradient was used: 0–15 min, linear 0–20% B; 15–20 min, linear 20–40% B; 20–20.5 min, linear 40–100% B; 20.5–20.6 min, linear 100–0% B; 20.6–24 min, isocratic 0% B. The volume of extract injected was 2 μL at a flow rate of 0.17 mL/min. The detection was recorded at 520 nm. All samples were injected in triplicate. The identification of anthocyanins was carried out in accordance with Braunlich et al. [27]. The anthocyanins quantification was performed using standards of delphinidin-3-O-galactoside (Del-3-Gal), cyanidin-3-O-galactoside (Cyn-3-Gal), cyanidin-3-O-glucoside (Cyn-3-Glu) and cyanidin-3-O-arabinoside (Cyn-3-Arb) prepared in methanol acidified with 1% HCl (*v*/*v*) in a concentration range from 6.25 to 200 μg/mL. The linear regression equations and the correlation coefficients (R^2^) of calibration curves were: y=6680x−20186 and 0.9978 for Del-3-Gal, y=15863x−169415 and 0.9944 for Cyn-3-Gal, y=8956x−5534.7 and 0.992 for Cyn-3-Glu and y=21412x−192469 and 0.9976 for Cyn-3-Arb. The cyanidin-3-O-xyloside was quantified as cyanidin-3-O-arabinoside. The results were expressed as milligram per gram of dry weight (mg/g dw). 

### 2.6. Cell Culture and Treatment

C2BBe1 cells, a clone of the Caco-2 cell line (CRL-2102 from the American Type Culture Collection) are enterocytes isolated from colon of a patient with colorectal adenocarcinoma. The C2BBe1 cells were grown in Dulbecco’s Modified Eagle’s Medium (Biowest, Nuaillé, France) supplemented with 0.01 mg/mL human transferrin (PanReac AppliChem ITW Reagents, Darmstadt, Germany), 1 × antibiotic-antimycotic solution 1 (Biowest, Nuaillé, France), 1.5 g/L sodium bicarbonate and 10% fetal bovine serum (Biowest, Nuaillé, France) in a 5% CO_2_ atmosphere at 37 °C. The establishment of the treatment doses and the cytotoxicity of the chokeberry and blueberry pomace extracts was performed by a MTT assay [4]. The C2BBe1 enterocyte cells were seeded at 2.5 × 10^5^/mL in complete medium in 12-well cell-culture treated multidishes (Nunc, Thermo Fisher Scientific, Roskilde, Denmark). After reaching approximately 80% confluence, the cells were exposed for 24 h to 0, 25, 50 and 100 mg/mL chokeberry pomace or blueberry pomace or their mix (1:1 *w*/*w*).

The pomace concentrations were prepared by diluting a 1:10 (*w*/*v*) aqueous extract of blueberry or chokeberry pomace. This involved suspending the pomace in water, ultrasonicating for 15 min at 35 kHz and 37 °C, centrifuging for 5 min at 5000× *g* and 4 °C, and filtering the supernatants through a cellulose filter. The resulting extract was then diluted to achieve the desired concentrations: a 1:2 dilution for a 50 mg/mL concentration and a 1:4 dilution for a 25 mg/mL concentration, using distilled water. For the combined blueberry and chokeberry pomace treatment, equal volumes of the 1:10 (*w*/*v*) aqueous extracts of each fruit pomace were mixed, followed by the same 1:2 and 1:4 dilutions. The cell culture media were prepared using the undiluted extract as well as the 1:2 and 1:4 dilutions. All prepared media were immediately sterilized by filtration through a sterile 0.22 µm pore diameter filter. The cultures were visualized after the 24 h treatment interval using the ZOE fluorescent cell imager, using the brightfield setting (Bio-Rad Laboratories, Hercules, CA, USA). After 24 h of exposure, the media was aspirated and the cells were washed with PBS. A volume of 500 μL of 1 mg/mL MTT solution was added to each well and the cells were incubated 2 h. The MTT solution was removed, and 500 μL isopropanol was added to dissolve the formazan crystals. The absorbance at 595 nm was read using a microplate reader (680 Microplate Reader, Bio-Rad Laboratories, Hercules, CA, USA).

Cell numbers and cell viability were assessed using a TC20 automated cell counter (Bio-Rad Laboratories, Hercules, CA, USA) and a trypan blue dye exclusion assay. Briefly, the cells were treated as in the case of the MTT test, but after the washing step with PBS a volume of 100 μL of 0.25% trypsin-EDTA (Biowest Nuaillé, France) was added to each well. After 4–5 min of incubation in a 5% CO_2_ atmosphere at 37 °C, 95% humidity, a double volume of complete culture medium was added over the detached cells and the cell suspension was collected in a 1.5 mL sterile tube. A volume of 20 μL cell suspension was homogenized with an equal volume of trypan blue dye and the mixture was introduced immediately in the counting chamber of the TC20 automated cell counter. Cell viability (%) was calculated as a ratio of the number of viable cells (unstained) to the total number of cells (unstained and stained).

After the establishment of the treatment doses (50 mg/mL pomaces extracts) the cells were sub-cultured in 25 cm^2^ flasks (Nunc EasYFlask, Thermo Fisher Scientific). At approximately 80% confluence, the cells were treated 24 h with pomace extracts of chokeberry, blueberry or mix (1:1 *w*/*w*) prepared as described above. For the subsequent studies, both the cells and the culture medium were collected.

### 2.7. Culture Medium and Cell Lysate Preparation

Culture medium from treated cells and controls was collected and used for subsequent LDH assay and cytokine assessment. Treated cells were detached using 0.25% trypsin-EDTA, the trypsin activity was inhibited by adding a double volume of complete medium before the cells were centrifuged and re-suspended in cold cell lysis buffer (provided by ReadyPrep Protein Extraction Kit (Membrane II), Bio-Rad Laboratories, Hercules, CA, USA). The cell suspensions were homogenized using ceramic beads and Bioprep 24R Homogenizer (Allsheng Instruments Co., Hangzhou, China) at 4 °C according to the manufacturer’s instructions. The lysate was placed on ice for 10 min and cell debris were removed by centrifugation at 14,000× *g* for 10 min at 4 °C. The protein content of the supernatants was determined using the Pierce Rapid Gold BCA Protein Assay Kit (Thermo Fisher Scientific, MA, USA). Finally, the supernatants were equalized at a concentration of 500 μg/mL, aliquoted and frozen at −80 °C, for subsequent biochemical and immunochemical analyses. The cell debris were removed by centrifugation at 10,000× *g* for 10 min at 4 °C. Finally, the supernatant was aliquoted and frozen at −80 °C for subsequent biochemical and immunochemical analyses.

### 2.8. Lactate Dehydrogenase Activity Evaluation in the Culture Medium

To evaluate cellular membrane integrity, the amount of lactate dehydrogenase (LDH) released into the culture medium was measured using the in vitro Toxicology Assay kit (Lactic Dehydrogenase) from Sigma (Sigma-Aldrich, St. Louis, MO, USA), as previously described [28]. The kit employs a colorimetric method that relies on the reduction of NAD+ by LDH, leading to the formation of a colored formazan derivative. The complete culture medium was collected and centrifugated at 10,000× *g* for 5 min at 4 °C to obtain a cell-free supernatant. The culture medium supplemented with 10% fetal bovine serum was used as a blank to correct for any background LDH activity. The corrected absorbance values were used to calculate the amount of LDH released into the medium. The LDH level is influenced by the number of cells present, and thus, the LDH activity was normalized to the total number of cells determined by the dye exclusion assay.

### 2.9. Inflammatory Cytokines and MMPs Quantifications

Collected culture medium and cell lysates were used for the detection of APRIL/TNFSF13, BAFF/TNFSF13B, sCD30/TNFRSF8, sCD163, CHI3L1, gp130/sIL-6Rβ, IFN-α2, IFN-β, IFN-γ, IL-2, sIL-6Rα, IL-8, IL-10, IL-11, IL-12 (p40), IL-12 (p70), IL-19, IL-20, IL-22, IL-26, IL-27 (p28), IL-28A/IFN-λ2, IL-29/IFN-λ1, IL-32, IL-34, IL-35, LIGHT/TNFSF14, MMP-1, MMP-2, MMP-3, Osteocalcin, Osteopontin, Pentraxin-3, sTNF-R1, sTNF-R2, TSLP and TWEAK/TNFSF12 biomarkers using the Bio-Plex Pro Human Inflammation 37-plex Panel 1 (Bio-Rad Laboratories, Hercules, CA, USA). The biomarker levels were analyzed according to manufacturer’s instructions, using the Bio-Plex MAGPIX System and Bio-Plex Manager software version 6.0 (Bio-Rad Laboratories, Hercules, CA, USA) as previously described [1].

### 2.10. Western Blot Assays

For target protein expression evaluation, 5 mg/well of whole cell lysates were resolved on Protean TGX Stain Free 4–20% precast gels (Bio-Rad Laboratories, Hercules, CA, USA), transferred onto 2 μm nitrocellulose membrane (V3 Western Workflow, Bio-Rad Laboratories, Hercules, CA, USA) and total protein transferred signal was detected and quantified using the ChemiDoc MP System and Image Lab software (version 5.2.1, Bio-Rad Laboratories, Hercules, CA, USA). The membranes were blocked using EveryBlot Blocking Buffer for 15 min at room temperature. Rabbit anti-human Erk1 (pThr202/pTyr204)/Erk2 monoclonal antibody (AHP2608, 1:1000 dilution factor), mouse anti-human OCLN monoclonal antibody (MCA3308Z, 1:250 dilution factor), mouse anti-human STAT1 monoclonal antibody (MCA3469Z, 1:350 dilution factor), goat anti-human STAT3 polyclonal antibody (AHP1076, 1:500 dilution factor) and mouse anti-human Akt-1 monoclonal antibody (MCA4779Z, 1:200 dilution factor) were used. HRP conjugated secondary antibodies STAR 207P (goat anti mouse IgG-HRP), STAR 121 P (goat anti rabbit IgG-HRP) and STAR 122 (rabbit anti goat IgG HRP) at 1:5000 dilution factor respectively, (Bio-Rad antibodies, Hercules, CA, USA) were used. For immunostaining of the membranes, they were incubated with the primary antibodies for 2 h and with the secondary antibodies for 1 h at room temperature, under constant homogenization. Blots were revealed using the Clarity Western ECL Substrate (Bio-Rad Laboratories, Hercules, CA, USA) and the chemiluminescence signal was detected using the ChemiDoc MP System. The target proteins expression was quantified using the Image Lab software version 5.2.1 (Bio-Rad Laboratories, Hercules, CA, USA), and normalized to the total proteins transferred onto the membrane (each protein band was normalized against the total proteins transferred in the corresponding lane) [4].

Cellular carbonylated protein detection was performed using the OxiSelect Protein Carbonyl Immunoblot Kit (Cell Biolabs, San Diego, CA, USA) with a post-transfer derivatization step of protein-bound carbonyl groups by treatment with a solution of dinitrophenylhydrazine (DNPH). The formed adducts were recognized by a primary rabbit anti-DNPH (diluted 1:1000), and HRP conjugated secondary antibody anti-rabbit IgG (diluted 1:1000) as previously described [4].

Aldehydic secondary products of lipid peroxidation such as malondialdehyde (MDA) and 4-hydroxynonenal are markers of oxidative stress that have been shown to be capable of binding to proteins and forming stable adducts, named advanced lipid peroxidation end products. The MDA-proteins adducts were detected using OxiSelect Malondialdehyde (MDA) Immunoblot Kit (Cell Biolabs, San Diego, CA, USA) that includes antibodies for the detection of MDA: Rabbit Anti-MDA Antibody (diluted 1:1000), and HRP conjugated anti-rabbit IgG secondary antibody (diluted 1:1000). 

### 2.11. Statistical Analysis

The variables used in this study are $x_{1}$—the concentration of polyphenols, $x_{2}$—the concentration of anthocyanins, y—the antioxidant activity, z—the absolute level of the analytes in the test cell cultures, $z_{0}$—the absolute level of the analytes in the control cell cultures, S—the survival in the cell cultures, c—the concentration of the extract used in the treatment of cell cultures. We also define the following a set of new variables through the ratio:

(1) $$w=\frac{z}{z_{0}}$$

which represents the value of an analyte in test cell cultures normalized to the value of the level of the same analyte in control cell cultures. As stated previously, the values of these variables were determined in triplicate, and the statistical tests and mathematical models described below refer to the mean values.

For the variables determined in the different phases of the experiment, the mean values, the standard deviation and the defined coefficient of variation were calculated:

(2) $$c_{v}=\frac{M}{SD}$$

mean values (M), standard deviation (SD). For comparisons, the *t*-Student test was applied and we calculated the difference between the means corresponding to the conditions that were compared (e.g., the antioxidant activity of the extracts obtained in different solvents, the antioxidant activity of the extracts obtained from different pomace fruits in the same solvent, the levels of the analytes normalized to levels obtained in control cultures or obtained by treating cell cultures with different types of extracts). The threshold value of statistical significance was considered (p=0.05).

### 2.12. Mathematical Models

Table 1 shows the hypotheses we checked in order to obtain a model which explains the antioxidant activity of the studied extracts with the maximum possible accuracy (i.e., minimal error) under the given conditions. In the model equations in Table 1, a, b are proportionality factors, while c represents the antioxidant activity due to other putative antioxidant species that are not quantified. To determine the factors a, b, c as well as the exponents, m, n we minimized using the Generalized Least Squares algorithm [29,30,31,32], the following quantity that represents the error of the models in Table 1:

(3) $$ER=\left(\sqrt{\frac{1}{N}\sum_{j}{\left[y_{e}^{\left(j\right)}-y_{e}^{\left(j\right)}\right]}^{2}}\right)$$

where $y^{\left(j\right)}$ is the value of the antioxidant activity according to one of the models, $y_{e}^{\left(j\right)}$ is the value determined in the experiment under the conditions (j), and N is the number of samples for which the oxidant activity was determined under different conditions. The antioxidant activity was determined for 2 types of berries: blueberries, chokeberries, from which extracts were obtained in 2 aqueous solvents and 3 ethanolic solvents (in total 5 extraction solvents), so N=10. We tested the models in the Table 1 by successively fixing m, n and letting the coefficients a, b, c vary. Then we allowed all these parameters to vary.

In the analysis of the effects of the extracts on cell cultures, we considered that the survival S (in percent, %) is an exponential decrease depending on the concentration of the extract, which has been applied on cell cultures:

(4) $$S=\alpha e^{-\beta c}+\gamma$$

The coefficients α, β, γ were obtained using the same optimization algorithm to minimize the model error (3):

(5) $$ER_{c}=\sqrt{\frac{1}{N_{c}}\sum_{k}{\left[S^{\left(k\right)}-S_{e}^{\left(k\right)}\right]}^{2}}$$

where $S^{\left(k\right)}\;$ is the survival according to the theoretical model (4) and $S_{e}^{\left(k\right)}$ is the value of the cell survival obtained experimentally when treating the cell culture with an identical concentration, c of berries extract, for which $S^{\left(k\right)}$ was calculated. It is also $N_{c}$ the number of cell cultures that have been treated with berries extracts. For each situation (blueberry, chokeberry, mixture of blueberries and chokeberries pomace extracts), the cell cultures were treated with 4 concentrations, respectively 0, 25, 50 and 100 mg/mL (in aqueous solvents), so $N_{c}=4$. Also, using the coefficients determined in this step, we calculated the concentrations for which the survival of cell cultures is 50% (denoted ${LD}_{50}$) by the formula:

(6) $${LD}_{50}=-\frac{1}{\beta }log\left(\frac{50-\gamma }{\alpha }\right)$$

## 3. Results

### 3.1. Chokeberry and Blueberry Pomace Extracts Characterization

To characterize the pomace extracts composition and to evaluate the efficiency of extraction parameters and solvents (50% ethanol, 70% ethanol, 100% ethanol, distilled water acidified with 0.5% vinegar and distilled water) in extracting total polyphenols content (TPC), total anthocyanins (TA) and antioxidant activity (AA), the results obtained via spectrophotometry and UPLC were calculated and then statistically analyzed. The mean values were then compared between the different samples to determine the potential optimal solvents and the differences in antioxidant properties between chokeberry and blueberry pomace extracts.

#### 3.1.1. Total Polyphenols Content

The effects of extraction solvents (50% ethanol, 70% ethanol, 100% ethanol, distilled water acidified with 0.5% vinegar and distilled water) at the same temperature and extraction time on TPC is shown in Table 2. The maximum levels of TPC were obtained mostly from samples extracted from chokeberry pomace with different proportions of ethanol in range of 47–51 mg GAE/g dw without significant statistical differences. The polyphenols extraction efficiency in distilled water in the presence or in absence of 0.5% vinegar was significantly lower (by 2.75-fold and respectively 5.5-fold, *p* < 0.001) than in the case of those performed in ethanol. However, it is observed that the acidification of distilled water improved the extraction of polyphenols or their stability in an aqueous environment, the TPC level obtained being almost double (Table 2). The blueberry pomace extracts gave significantly lower (*p* < 0.001) TPC yield compared with chokeberry pomace extracts, the highest TPC yield being obtained in the case of alcoholic extracts. For example, in the case of the extracts in 50% ethanol, chokeberry had a TPC approximately 4.8-fold higher compared the TPC of aqueous extract (*p* < 0.001) (Table 2). As in the case of chokeberry, blueberry extracts showed no significant differences in the level of TPC between the extracts obtained in 50 and 70% ethanol, but using 100% ethanol, the yield of polyphenols decreased by 2.75 folds (*p* < 0.001), being similar to that of extracts in distilled water (*p* > 0.05). Acidification with vinegar had no effect on the TPC level in the case of the blueberry pomace extracts. But compared to the chokeberry pomace extracts in distilled water, those from blueberry had a TPC content approximately 2.4-fold lower. In addition, it was observed that polyphenols extraction from chokeberry was more efficient in 50–70% ethanol compared to blueberry extracts.

#### 3.1.2. Anthocyanins Composition

The total anthocyanin content and anthocyanin components of chokeberry and blueberry SPE purified extracts were identified by ultra-performance liquid chromatography-mass spectrometry-UV/VIS Diode Array Detector (UPLC-UV/VIS PDA) analysis (Table 3, Figures S1 and S2). As for the chokeberry anthocyanins, they were mainly composed of cyanidins, which combine different glycosides (Figure S1). The main anthocyanins we identified and quantified were cyanidin-3-O-galactoside and cyanidin-3-O-arabinoside, which reached the highest level in 50% ethanol extract (2.10 and 0.61 mg/g dw from a total of 3.05 mg anthocyanins/g dw, Table 3). No significant differences were recorded in the content of anthocyanins between chokeberry pomace extracts performed in 70% ethanol and 50% ethanol. Significant differences in the total content of anthocyanins as well as the percentage composition were recorded between the alcoholic and aqueous extracts, the latter registering the lowest level of anthocyanins of 1.58 mg/g dw (Table 3). In the aqueous pomace chokeberry extracts the percentage of cyanidin-3-O-galactoside was higher compared to those recorded in alcoholic solutions, being probably more easily soluble in water acidified with 0.5% vinegar than the other anthocyanins.

The blueberries SPE purified extracts chromatogram profiles is provided in Figure S2. The separation profiles showed seven distinct peaks at retention times of 4.47, 4.96, 5.22, 5.47, 5.76, 5.90 and 6.42 min. Based on the available standards, we could only confirm Del-3-O-Gal, Cyn-3-O-Gal and respectively Cyn-3-O-Glu at retention times 4.47, 4.96 and 5.22 min, respectively. The other peaks probably belong to the other major anthocyanins such as malvidin glycoside, petunidin glycoside and peonidin glycoside detected by other authors [18,19,33,34]. Surprisingly, despite the notably higher levels of polyphenols in chokeberry extracts compared to blueberry extracts (Table 2), the total anthocyanin levels in the 50% alcoholic extracts showed no significant differences between the two berries (Table 3). However, in the 70% ethanol extracts, the anthocyanin levels from blueberry pomace were significantly higher (*p* < 0.05, Table 3). In contrast, in vinegar-acidified water, the total anthocyanin levels determined from chokeberry pomace extracts were significantly higher than those determined for blueberries (*p* < 0.001, Table 3).

#### 3.1.3. Antioxidant Activity

Table 4 presents the results of the antioxidant activity tests conducted on chokeberry and blueberries pomace extracts. The findings showed that the ethanolic extracts have stronger antioxidant activity, as measured by DPPH radical method, compared to the water extracts in both pomaces. However, chokeberry extracts exhibit significantly higher antioxidant activity compared to blueberries (*p* < 0.001). There was no noticeable difference in the antioxidant potential between chokeberry 50 and 70% ethanolic pomace extracts. Additionally, there were no significant differences in the antioxidant activities between the aqueous extracts of blueberries. In contrast, the antioxidant capacities of the aqueous extracts of chokeberry were more than four times higher than those recorded for blueberries (*p* < 0.001). 

#### 3.1.4. Mathematical Models: Evaluation of the Dependence of Antioxidant Activity on Polyphenol and Anthocyanin Levels

Table 5 shows the coefficients obtained by applying the optimization algorithm supposing successively that hypotheses H1–H3 from Table 1 are true. We note that in the case of H1, where we considered the antioxidant activity exclusively dependent on polyphenols, the linear model (H1.1) has led to the smallest error. A similar error was also obtained by optimizing the H1.4 model, where the power of the term $x^{m}$ is m=0.93 (very close to the value corresponding to the H1.1 model). Thus, within the H1 hypothesis, there was a linear dependence of antioxidant activity on the concentration of polyphenols. Among the models underlying hypothesis H2, model H2.4, which describes a fractional power dependence on the concentration of anthocyanins (n=0.24), was the model with the smallest error. Within this model, c=0.00, which is interpreted as a negligible contribution of other unquantified species to the antioxidant activity. The linear model H2.1 was optimized for negative values of b, which is in contradiction with the conditions in Table 1. A negative value of the coefficients a and b would imply pro-oxidant effects of the studied species, which obviously does not correspond to reality. Among the H3 models, model H3.3, which assumes a linear dependence on the concentration of polyphenols (without anthocyanins) $x_{1}-x_{2}$ and cubic on the concentration of anthocyanins $x_{2}$, was the model for which optimization yielded the smallest error. Two other models also yielded small errors (3.24 and 3.79 larger than the error of model H3.3). Models H3.4 and H3.5 also yielded similar results. In these two models, the coefficients a and b had values very close to 0, which almost cancels the contributions of the two classes of compounds to antioxidant activity. This situation does not correspond to reality, so these two models were not considered. 

We observe that as our models became more nuanced (from the exclusive dependencies described by models H1 and H2 to the more comprehensive forms of H3), the errors of the models reaching the smallest values for the latter class of models, leading to the conclusion that, among the species with antioxidant role, anthocyanins are the most potent antioxidants, their effective contribution being quantified by a cubic function.

### 3.2. Antiproliferative and Cytotoxic Effects of Chokeberry and Blueberry Fruit Pomace

**Figure 1:**
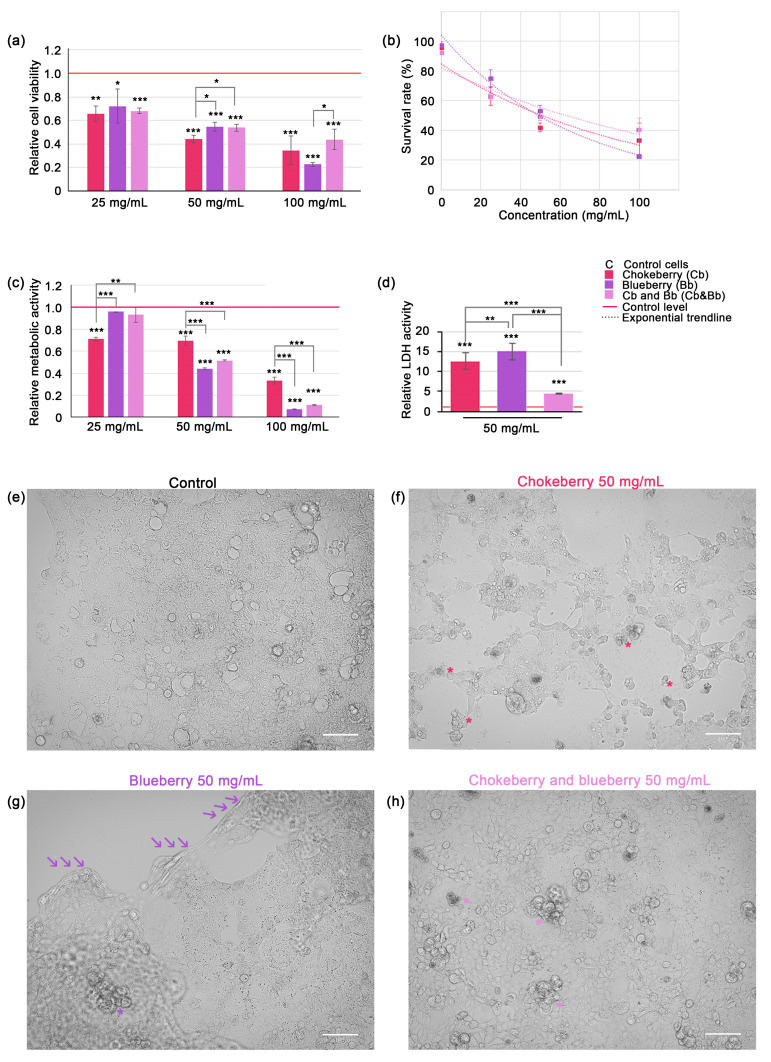
Assessment of C2BBe1 cell viability and proliferation under chokeberry or blueberry pomace treatment and microscopy observations. (**a**) Relative cell viability across varying concentrations of Cb or Bb or both; (**b**) Dose-response curves representing the exponential inverse relationship between cell viability and fruit pomace concentrations; (**c**) Relative metabolic activity in C2BBe1 cells exposed to Cb, Bb or both fruit pomace varying concentrations (MTT assay); (**d**) Relative LDH activity in cell culture media. All graphs represent mean ± SD, and statistically significant differences are marked by * *p* < 0.05, ** *p* < 0.01, *** *p* < 0.001; (**e**) brightfield micrograph of control C2BBe1 cells in culture (**f**) brightfield micrograph of cultured C2BBe1 cells after 24 h exposure to 50 mg/mL chokeberry pomace aqueous extract. Numerous apoptotic bodies (*), cell debris and cell shrinkage can be observed; (**g**) brightfield micrograph of cultured C2BBe1 cells after 24 h exposure to 50 mg/mL blueberry pomace aqueous extract. Cell monolayer is detaching from the substrate (arrows) while a few apoptotic bodies can be observed (*), along with a notable swelling of the cells still attached. (**h**) brightfield micrograph of cultured C2BBe1 cells after 24 h exposure to 50 mg/mL chokeberry and blueberry pomace mix aqueous extract. Several apoptotic bodies (*) can be observed, with no cell detachment from the substrate or swelling. Bar represents 100 µm.

The effect of chokeberry and blueberry pomace on the C2BBe1 colorectal carcinoma cell viability and proliferation was assessed using the trypan blue dye exclusion and MTT assays. Both chokeberry and blueberry extracts and their mixture diminished cell viability significantly, in dose dependent manner. The highest decreases were registered with blueberry and chokeberry independent treatment at the 100 mg/mL dose (0.35 and 0.23-fold respectively compared to control level, *p* < 0.001). The treatment using chokeberry and blueberry pomace mixture extracts reduced cell viability in a lesser extent, and reached at the 100 mg/mL dose 0.44-fold of control level, *p* < 0.001 (Figure 1a). Only in the case of blueberry pomace extract we did identify a significant change when comparing the cell viability at this dose with the one registered at the 50 mg/mL dose (*p* = 0.00017), suggesting the reduction of cell viability had closer dependency to the dose in this case. 

Based on cell viability data we plotted the dose-response curves (see Equation (4) and Figure 1b). The LD50 doses for 24 h were calculated as 52.80 mg/mL for chokeberry pomace, 48.98 mg/mL for blueberry pomace and 62.32 mg/mL for chokeberry and blueberry pomace mixture extracts. This data suggests that in our experimental setup the blueberry pomace was the most powerful inhibitor of cell viability. For all other experiments (except MTT assay) we used only the 50 mg/mL pomace dose, which was closest to LD50 for both chokeberry and blueberry pomaces. MTT assay confirmed that blueberry pomace was also the most potent inhibitor of cell proliferation, as relative cell metabolism decreased markedly to 0.07-fold of control level (*p* < 0.001) (Figure 1c). Contrary to the cell viability data, which indicated that the pomace mixture was the least potent inhibitor of cell viability, the mixture of chokeberry and blueberry pomace was the second most powerful inhibitor of cell proliferation. At the 100 mg/mL dose, the mixture reduced cell proliferation to 0.11-fold of the control level (*p* < 0.001), a level not statistically different from that achieved by blueberry pomace alone at the same dose. This parity in cell proliferation inhibition was also observed at the lower doses of 50 mg/mL. It is important to note that at the 25 mg/mL dose, neither the blueberry pomace nor the pomace mixture showed a significant reduction in cellular metabolism relative to the control, which was unexpected given the significant reduction in cell viability under the same conditions. Based on these observations, we suggest that the 25 mg/mL dose of chokeberry pomace was more effective at reducing cellular proliferation than the other two treatments, while all pomaces at this dose exhibited significant cytocidal effects. The pomace extracts induced significant plasma membrane leakage in the colorectal carcinoma cells, as evidenced by a substantial increase in extracellular LDH activity. After 24 h of exposure to 50 mg/mL blueberry pomace caused a 15-fold increase, and the same dose of chokeberry pomace caused a 12.6-fold increase in LDH activity (*p* < 0.001). Interestingly, the mixture of chokeberry and blueberry pomace caused a comparatively lower increase in LDH activity, with a 4.35-fold rise relative to the control level (Figure 1d). This increase was statistically significant relative to control cells (*p* < 0.001), however it was significantly lower than the increase induced by chokeberry pomace alone (*p* < 0.01) and blueberry pomace alone (*p* < 0.001). 

Microscopic observations confirmed that cell death was induced following 24 h of exposure to chokeberry and blueberry pomace extracts at a concentration of 50 mg/mL. The typical morphology of C2BBe1 cells, a clone derived from Caco-2 cells, is depicted in Figure 1e. Under our experimental conditions, C2BBe1 cells formed heterogeneous monolayers characterized by polygonal or epithelioid shapes with varying levels of cytoplasmic granularity and irregular cell borders. The cells displayed prominent nuclei, some of which were irregularly shaped or multi-lobed, indicative of their malignant nature. We selected this particular cell line as a model due to its relevance in studying the potential therapeutic effects of polyphenols and anthocyanins extracted from chokeberry and blueberry pomace against colorectal cancers. The exposure to 50 mg/mL chokeberry pomace induced changes very evident under brightfield microscopy, such as cell shrinkage, cytoplasmic and nuclear changes indicative of condensation, the formation of apoptotic bodies (indicated by * in Figure 1e), alterations classically associated with apoptosis. Numerous cellular debris were present. Similar changes, albeit to a much lesser extent were noted in the cells exposed to the pomace mixture (apoptotic bodies indicated with * in Figure 1h). Exposure to blueberry extract, however, did not elicit similar morphological changes, with apoptotic bodies rarely visible (* in Figure 1g). Instead, cells within the monolayer exhibited noticeable swelling prior to detachment in sheets from the culture flask surface. The region of detachment is demarcated by a series of three arrows in Figure 1g. 

### 3.3. Antioxidative Effects of Chokeberry and Blueberry Pomace Extracts

**Figure 2:**
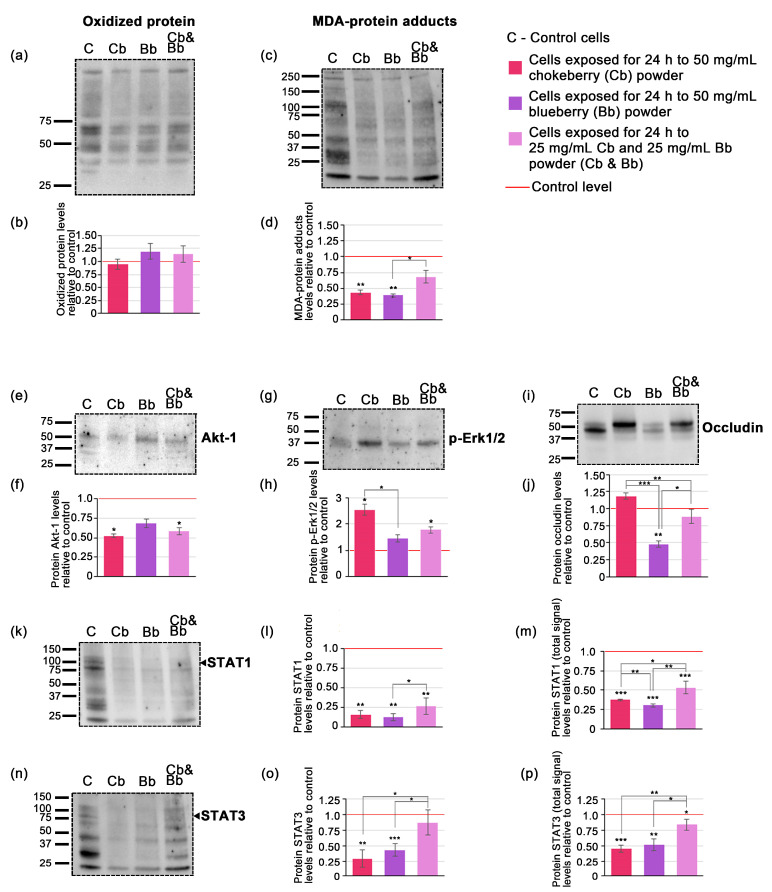
Western blots and the densitometric analysis (**a**) blotted membrane example corresponding to oxidized protein; (**b**) densitometric analysis of blotted membranes corresponding to oxidized proteins; (**c**) blotted membrane example corresponding to MDA-protein adducts; (**d**) densitometric analysis of blotted membranes corresponding to MDA-protein adducts; (**e**) blotted membrane example corresponding to Akt-1; (**f**) densitometric analysis of blotted membranes corresponding to Akt-1; (**g**) blotted membrane example corresponding to p-Erk1/2; (**h**) densitometric analysis of blotted membranes corresponding to p-Erk1/2; (**i**) blotted membrane example corresponding to occludin; (**j**) densitometric analysis of blotted membranes corresponding to occludin (**k**) blotted membrane example corresponding to STAT1 (**l**) densitometric analysis of blotted membranes corresponding to STAT1 at specific molecular weight; (**m**) densitometric analysis of blotted membranes corresponding to STAT1 total signal (**n**) blotted membrane example corresponding to STAT3; (**o**) densitometric analysis of blotted membranes corresponding to STAT3 at specific molecular weight (**p**) densitometric analysis of blotted membranes corresponding to STAT3 total signal. Graphs represent relative fold changes of protein expression, and are expressed as means ± SD. Statistically significant differences are marked by * *p* < 0.05, ** *p* < 0.01, *** *p* < 0.001. The uncropped blot images as well as images of SDS-PAGE gels and transferred membranes are available in Figure S3.

Detection of oxidized proteins and MDA-protein adducts serves as valuable markers for assessing the antioxidative effects of compounds present in pomace extracts. In presence of reactive oxygen species (ROS) proteins are susceptible to oxidation through interactions with ROS or via interaction with lipid peroxidation products like malondialdehyde (MDA). Therefore, a reduction in oxidized proteins and protein-MDA adducts following treatment with antioxidant-rich pomace compounds suggests antioxidative properties. Western blot analysis detected signals specific to oxidized proteins and MDA-protein adducts across a range of molecular weights, from approximately 37 kDa to 250 kDa for oxidized proteins (Figure 2a) and from 25 kDa to 250 kDa for MDA-adducts (Figure 2c). Each signal exhibited distinct banding patterns, distinguishing oxidized proteins from MDA-protein adducts. 

Densitometric analysis of the Western blot data indicated that the levels of oxidized proteins in whole cell lysate extracts did not significantly differ from the control under all conditions tested. Specifically, chokeberry treatment showed a modest 0.95-fold decrease in oxidized proteins, whereas both blueberry and the pomace mixture demonstrated slight increases; however, these changes were not statistically significant (Figure 1b). The level of MDA-protein adducts decreased significantly only in the case of individual blueberry and chokeberry pomace exposure, by 0.39-fold and 0.44-fold (*p* < 0.01), while the pomace mixture only reduced MDA-protein adducts by 0.68-fold (*p* = 0.06) (Figure 1d).

### 3.4. Evaluation of Potential Signaling Mechanisms Affected by Cells Exposure to Chokeberry and Blueberry Pomace Extracts

In order to elucidate the potential signaling mechanisms affected by exposure to the chokeberry and blueberry pomace extracts we assessed the protein expression levels of a quartet of relevant signaling proteins, namely Akt-1, p-Erk1/2, STAT1 and STAT3. Western blot analysis revealed specific bands for Akt-1 (Figure 2e), p-Erk1/2 (Figure 2g) and occludin (Figure 2j) at their expected molecular weights indicated in the primary antibody datasheets. STAT1 and STAT3 blots exhibited chemiluminescent signals at multiple molecular weights, displaying distinctive band patterns (Figure 2k,n). Consequently, in these cases, densitometric analysis was conducted for the bands corresponding to the expected molecular weights of each protein based on the primary antibody datasheets, as well as for all the bands within each lane, which we designated as the total signal for STAT1 and STAT3. This approach was deemed useful as recent research highlights the existence of multiple protein isoforms of both STAT1 and STAT3 in tumoral cells [35,36]. Following a 24-h cells exposure to 50 mg/mL of chokeberry or chokeberry-blueberry pomace mixture, the protein expression level of Akt-1 decreased by 0.52-fold and 0.58-fold, respectively, compared to the control (*p* < 0.05). In contrast, the relative protein expression level of p-Erk1/2 increased by 2.55-fold under chokeberry pomace treatment, and by 1.78-fold with the chokeberry and blueberry pomace mixture (both with *p* < 0.05). The blueberry pomace extract treatment did not induce significant changes in neither Akt-1 nor p-Erk1/2, the former demonstrating a 0.68-fold decrease and the latter a 1.45-fold increase relative to controls. STAT1 and STAT3 protein expression levels diminished under all pomace treatments, with both their expected molecular weights and total STAT displaying similar profiles (Figure 2l,m,o,p). Notably, with regard to STAT expression, both chokeberry pomace and blueberry pomace had comparable effects, with STAT1 decreasing by 0.16-fold and 0.12-fold respectively (Figure 2l) and STAT3 diminishing by 0.30-fold and 0.44-fold respectively (Figure 2o). These changes were significantly more consistent than those induced by the pomace mixture which only reduced STAT1 by 0.26-fold and STAT3 by 0.88-fold compared to control (Figure 2l,o). The occludin protein expression profile showed the variable changes among the treatments (Figure 2i,j). The most consistent and significant modification was induced by the blueberry pomace treatment, which resulted in a 0.48-fold decrease (*p* < 0.01). The other treatments did not induce significant changes, with chokeberry pomace treatment leading to a 1.18-fold increase while the pomace mixture decreased occluding protein levels by 0.89-fold compared to the control. 

### 3.5. Cytokine Assessment and Inflammatory Response

To assess the capacity of chokeberry, blueberry, and combined pomace extracts to modulate an inflammatory response, we tested the expression levels of a panel of 37 cytokines using a magnetic bead-based assay. Among the 37 cytokines, 10 were detected at measurable levels in whole cell lysates (Table 6), and 11 were detected in the cell culture media (Table 7). We were able to detect chitinase-3-like 1, gp130, IL-8, IL-32, IL-35, LIGHT (TNFSF14), MMP-1, MMP-2, MMP-3 and TSLP in both cell lysates and culture media, while sTNFR1 was present at detectable levels only in the cell culture media. Overall, blueberry pomace ranked first when considering its ability to induce changes, in the expression of these inflammatory markers, with the pomace mixture being ranked second, and the chokeberry pomace last. Notably, the pomace mixture and chokeberry pomace sometimes had similar effects (MMP-1 and MMP-3 in cell lysates (Table 6) and chitinase-3-like 1 and gp130 in cell culture media (Table 7). Within the inflammatory markers we distinguished two groups: those that exhibited an increase and those that exhibited a decrease following the pomace treatments. The cytokines TSLP, LIGHT, and IL-35 exhibited increased expression compared to the control. Notably, the blueberry pomace treatment resulted in the most significant augmentation, increasing by 4.8-fold, 3.2-fold, and 2.7-fold in cell lysates, and by 9.2-fold, 4.2-fold, and 3.6-fold in cell culture media, respectively.

Among the inflammatory markers whose expression diminished, the most affected were Chitinase-3-like 1 and IL-8 in cell lysates, and Chitinase-3-like 1, gp130, IL-8, MMP-1, and sTNFR1 in cell culture media. The blueberry pomace extract treatment tended to induce the most substantial decreases, with Chitinase-3-like 1 in whole cell lysates and gp130 in cell media both decreasing to 0.25-fold of the control. Conversely, gp130 in cell lysates and MMP-3 in cell culture media showed the least reduction in expression.

## 4. Discussion

The chokeberry and blueberry fruit pomaces which are rich in polyphenols make them important candidates for the development of functional food formulations and nutraceuticals with a wide range of pharmacological effects, including antiproliferative effects [21,37,38,39,40,41], antioxidant effects [33,42,43], anti-inflammatory effects [9,33,44], hypoglycemic and anti-obesity effects [9,45] as well as the prevention and treatment of neurodegenerative and cardiovascular diseases [15,46]. The concentration of polyphenolic compounds depends on the cultivar variety, cultivation conditions, and ripening at harvest time and extraction method. While alternative extraction methods like ultrasound-assisted, pulsed electric field-assisted, microwave-assisted, and supercritical fluid extraction are known for their high efficiency, the conventional solvent extraction methods is straightforward and cost-effective [11], and by employing it we achieved substantial isolation of bioactive compounds from blueberry and chokeberry pomaces.

To evaluate and compare the antioxidant and antiproliferative effects of chokeberry and blueberry pomace extracts, we first conducted a comparative analysis of the total polyphenol content, anthocyanin composition, and antioxidant capacity using various solvents (50% ethanol, 70% ethanol, 100% ethanol, distilled water acidified with 0.5% vinegar, and distilled water). Regardless of the solvent used, the chokeberry pomace extracts had a higher content of total polyphenols and antioxidant activities compared to blueberry pomace extracts (Table 2 and Table 4). Interestingly, for the two berries, the extracts in 50% and 70% alcohol showed no significant differences in total polyphenol content (TPC), antioxidant activity (AA), or total anthocyanins (TA), except in the case of the blueberry alcohol extracts (Table 2, Table 3 and Table 4). In a recent study conducted by Kavela et al. [47], which investigated the efficiency of green solvents and parameters for the extraction of polyphenols from chokeberry pomace, the authors showed that 50% ethanol + 1% citric acid resulted in the highest yields of TPC and TA, followed by 50% glycerol + 1% formic acid, while 100% water gave the lowest yields under all extraction conditions. Thus, extraction in 50% ethanol + 1% citric acid for 60 min at 50 °C resulted in 74.96 mg GAE/g dw (TPC) and 13.13 mg TA/g dw, while in water + 1% citric acid, 27.12 mg GAE/g dw TPC and 8.46 mg TA/g dw were recorded [47]. Although the TPC and TA levels registered in our study were obtained at 37 °C (to simulate human body temperature) for 15 min, the TPC levels in 50% ethanol and in 0.5% vinegar water solution were only 1.5 times lower in both conditions and the TA levels were 4.3 times and 5.3 times lower, respectively. Extracting for a shorter period can prevent the degradation of polyphenols that may occur with longer extraction times. In addition, although elevated temperatures can help maximize yield, they can also negatively impact the yield of polyphenols and in organism, the temperature is 37 °C. The conditions used in the present study align with Le et al., who reported a decline in anthocyanin concentration as the temperature rises above 50 °C [48]. In another study conducted on chokeberries pomace, the TPC level was lower than that recorded in our study, specifically 27.99 mg GAE/g dw, even though the extraction was performed in 80% methanol for 24 h on a rotary shaker, and the extract exhibited an antioxidant activity of 400 mmoles TE/kg [6]. In contrast, our alcoholic extracts averaged 660 mg TE/g dw, indicating that the extraction conditions chosen in this study did not negatively impact the level or antioxidant activity of polyphenols. Additionally, the extraction time should be relatively short and the temperature low, as anthocyanins are sensitive to heat and degrade when exposed to high temperatures for extended periods [19]. 

The anthocyanin composition of chokeberry pomace extracts obtained through UPLC separation was consistent with the results from other studies on chokeberry pomace [47,49]. However, their results, in terms of total anthocyanin levels quantified as the sum of individual separated anthocyanins, are slightly higher than those obtained in this study. This discrepancy can be attributed to several factors, including the extraction method and the specific cultivar used. The UPLC patterns observed in this study are consistent with those reported by several other studies [47,49,50], where cyanidin-3-galactoside eluted first and exhibited a higher peak than the other cyanidins (Figure S1). All pomace extracts analyzed indicated a high yield of cyanidin-3-galactoside ranging as percentage from 64.4 to 75% (Table 3). 

In a study focused on the polyphenol composition and antioxidant capacity of blueberries at different ripening stages, using similar processing conditions except for the extraction solvent, which was a mix of water and formic acid extraction solution (40:40:20:0.1, *v*/*v*), it was demonstrated through HPLC that the total polyphenol level ranged between 8.39 and 13.35 mg/g dw, and the antioxidant activity of ripe fruits was approximately 125 mg TE/g dw [51], results comparable to those recorded in our study (Table 2 and Table 4). These authors successfully separated and quantified eight anthocyanins using HPLC in the following percentages: 10.4% cyanidin-3-glucoside, 12.0% cyanidin-3-galactoside, 17.0% delphinidin-3-galactoside, 14.3% delphinidin-3-glucoside, 20.5% petunidin-3-glucoside, 15.4% petunidin-3-galactoside, 6.0% malvidin-3-glucoside, and 4.3% malvidin-3-galactoside, totaling 5.65 mg TA/g dw [51], a slightly higher level than that found in our study (Table 3). However, the levels of cyanidin-3-glucoside, cyanidin-3-galactoside, and delphinidin-3-galactoside are quite similar to those recorded in the present study. Comparable results regarding polyphenol composition and antioxidant capacity have also been obtained by other authors in their studies [19,34].

Interestingly, in most cases, the anthocyanin fraction’s contribution to the total antioxidant activity of plant phenolic extracts and matrices was more significant compared to the total crude extracts. This suggests a high-impact biological and nutraceutical value of these compounds in plants and foods [15,20,21], as highlighted in our study through mathematical modeling (Table 5).

As shown in Table 3, the anthocyanin levels do not differ significantly in the alcoholic extracts of the two berries, while in water, the level in aronia was 1.9 times higher. However, the blueberry extract had a more pronounced effect on cell viability, proliferation, and cytotoxicity (Figure 1). This may be due to the different anthocyanin profiles of the two berries, with chokeberry containing only cyanidin-3-glycoside, while blueberries having a high percentage of other anthocyanins such as delphinidin-3-glycoside, petunidin-3-glycoside, and malvidin-3-glycoside.

While different extraction solvents were evaluated for their efficiency, our primary focus was on the biological effects of aqueous extracts of chokeberry and blueberry pomace, which prevented potential interference from ethanol contamination on cell cultures and also reflects conditions present in foods that may incorporate these pomaces in the future. 

**Figure 3:**
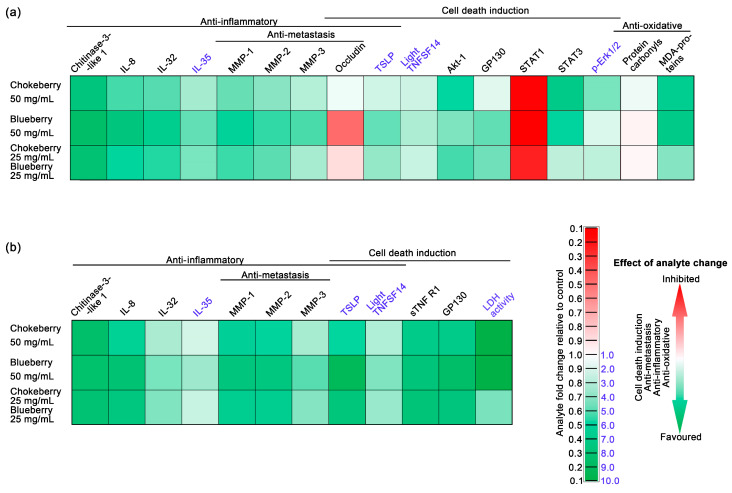
The effects of chokeberry, blueberry, and their pomace mixture on C2BBe1 colorectal cancer cells using an integrative heat-map style. The assessed parameters are represented as fold changes compared to controls and are color-coded to indicate the overall outcome of the changes. Green represents beneficial changes, such as the induction of cell death in the cancer cells, antimetastatic effects, anti-inflammatory effects, and antioxidative effects. (**a**) Parameters assessed in whole-cell lysates; (**b**) Parameters assessed in cell culture media.

The anti-inflammatory properties of blueberry and chokeberry hold particular importance in diseases marked by elevated inflammatory responses and oxidative stress [52,53,54], with cancer being a prime example. Understanding their anti-inflammatory properties is crucial in cancer research, as inflammation profoundly influences tumor development and therapeutic outcomes [55]. The anti-inflammatory properties of blueberry and chokeberry may play a pivotal role in controlling the tumor microenvironment and may potentially enhance treatment efficacy. Among the inflammatory marker panel we analysed chitinase-3-like 1 [1,56], IL-8 [57,58] and IL-32 [59,60] expression contributes to pro-inflammatory environment and are associated with an overall negative health outcome while the expression of IL-35 is considered an inflammatory dampener being associated with overall positive health outcomes for colon cancer patients [61,62]. As depicted in the integrative Figure 3, in which we employed color coding (green for favorable changes and red for unfavorable ones), the expression of chitinase-3-like 1, IL-8 and IL-32 in cells exposed to either chokeberry pomace, blueberry pomace or a mixture of these two was decreased relative to untreated cells, with the lowest levels being those under blueberry pomace treatment (See also Table 6 and Table 7). To our knowledge, this study represents the first documentation of the inhibitory effect of these widely recognized anti-inflammatory foods on chitinase-3-like 1 expression. 

Our previous research has identified chitinase-3-like 1 to be a cytokine with notably elevated protein expression levels in colorectal cancer tumors compared to normal colon tissues [1]. Chitinase-3-like 1 overexpression in colorectal cancer cells in vitro was shown to upregulate IL-8 secretion and activate Erk1/2 and JNK [63]. Moreover, the cytokine was also shown be involved in MMP-2 overexpression [64]. In addition, our previous research has contributed to strengthen this chitinase-3-like 1—pErk1/2—MMP-2 triad, indicating positive correlations between chitinase-3-like 1—pErk1/2 and chitinase-3-like 1—MMP-2, to be central in the development of local and distal tumors [1]. The inhibition of IL-8 observed in our study contrasts with previous research, which showed that a chokeberry bioactive fraction inhibited IL-8 gene and protein expression in bronchial epithelial cells only when challenged by LPS. In the absence of a pro-inflammatory stimulus, such as LPS, Jang et al. did not observe significant changes in IL-8 expression [65]. Our findings indicate that chokeberry bioactive compounds can inhibit IL-8 expression without the need for an external pro-inflammatory challenge. Moreover, our findings suggest that compounds from blueberry, even more so than chokeberry, inhibit IL-8 expression in unstimulated Caco-2 enterocytes, suggesting they could modulate the tumor microenvironment and inhibit cancer progression (Table 6 and Table 7, and Figure 3). Taverniti et al. provided evidence supporting an upstream regulatory mechanism that could explain the decreased expression of pro-inflammatory cytokines through NF-κB signaling inhibition by an anthocyanin-rich fraction of wild blueberry in Caco-2 cells [66]. Our own previous research supports NF-kB expression is paramount for pro-inflammatory cytokine and MMP expression in Caco-2 cells [4]. In the current study, we found decreased expression of pro-inflammatory IL-8 cytokine and MMPs-1, -2 and -3, under chokeberry and blueberry pomace treatments, supporting the idea that NF-kB signaling may be inhibited by the bioactive compounds in the pomace extracts. 

LIGHT, a member of the tumor necrosis factor superfamily holds promise for advancing cancer immunotherapy treatment strategies and has been under pre-clinical development [67,68]. The upregulation of LIGHT under chokeberry and blueberry pomace treatment in C2BBe1 colorectal cancer cells represents a novel finding (Table 6 and Table 7, and Figure 3), indicating that the bioactive compounds from these fruits may have a potential use as adjuvants in cancer therapy. Blueberry pomace exhibited the most consistent enhancement of LIGHT expression, particularly notable in the cell culture media, suggesting the potential for release from cancer cells. This hypothesis warrants further investigation to elucidate the mechanisms underlying this phenomenon. 

Matrix metalloproteinases (MMPs) are pivotal regulators of inflammation, primarily functioning as proteolytic enzymes that degrade the extracellular matrix [69]. Their significant roles in tumorigenesis and metastasis underscore their importance in cancer research, including colon cancer. Tumor cells often show increased levels of multiple MMPs, among which MMP-1, -2 and -3 are significantly associated with colon adenocarcinomas [1], particularly higher expression levels of these MMPs were also correlated with worse clinical outcomes that impact the survival of patients diagnosed with colon adenocarcinoma [70,71]. In line with this, we interpret the reductions in the protein levels of MMP-1, -2, and -3 following all the pomace treatments as having potential positive implications in colorectal cancer (Table 6 and Table 7, Figure 3). Our results are in agreement with previous studies indicating MMPs inhibition by chokeberry bioactive compounds in glioblastoma cells [72] and blueberry in prostate cancer cells [73]. In our experimental conditions, blueberry pomace extract most effectively inhibited MMP-1, MMP-2, and MMP-3 in C2BBe1 colorectal cancer cells (Table 6 and Table 7). 

The loss of occludin can disrupt tight junction integrity and lead to the loss of cohesion in cellular structures, facilitating the invasion and metastasis of cancer cells [74]. Research on the effects of blueberry and chokeberry on occludin expression and the integrity of tight junctions is not conclusive. For instance, Valdez et al. [75] demonstrated that chokeberry treatment effectively countered intestinal barrier dysfunction in Caco-2 cells under pro-inflammatory conditions by upregulating occludin expression levels, as demonstrated in our study. Conversely, recent findings by Marino et al. demonstrated that while wild blueberry treatment enhances the barrier function of tight junctions in Caco-2 cells, this effect does not involve the modulation of occludin expression [76]. In accordance with prior research, occludin expression appears to be influenced by specific conditions, including the combined effects of the extract’s anthocyanin composition, dosage, and the antioxidative properties of the biological source. In our study, exposure to 50 mg/mL blueberry pomace significantly reduced occludin levels, which aligns with our microscopic observations showing substantial cell detachment from the growth flask substrate (Figure 1g). When considered alone, the reduction of occludin might appear to promote metastasis. However, under our experimental conditions, cell viability was notably reduced, and the detached cells were mostly either dead or swollen, suggesting impending necrosis (Figure 1), suggesting a low likelihood of metastatic spread, though further study is necessary to confirm this. The anti-apoptotic role of lower occludin expression has been highlighted in a previous study, which demonstrated that occludin is essential for apoptosis when claudin-claudin interactions are disrupted [77]. Additionally, the mislocalization of occludin and claudin has been observed in many epithelial-derived tumors [78], likely contributing to their resistance to apoptosis. Thus, we can infer that occludin inhibition by blueberry pomace may have an anti-apoptotic effect. This may explain the distinct morphologies observed: apoptosis-like in cells exposed to chokeberry pomace and necrosis-like morphology in cells exposed to blueberry pomace (Figure 1f,g). 

This study highlighted a significant dose-dependent cytotoxicity of the aqueous extracts of chokeberry pomace, blueberry pomace, and their mixture on C2BBe1 cells (Figure 1). This is consistent with previous reports indicating chokeberry toxicity on bronchial epithelial cells [65], in an established glioblastoma cell line [72], and in HT-29 colon cancer cells [40], while wild blueberry extracts were shown to be cytotoxic to Caco-2 cells, reducing both cell viability and metabolic activity [76] and the phenolic fraction in blueberries induced apoptosis in HT-29 and Caco-2 colon cancer cells [41]. Recently, Wei et al. proposed that anthocyanins derived from chokeberry can interfere with the Wnt/β-catenin signaling pathway and trigger apoptosis in Caco-2 cells [39]. 

Elevated levels of extracellular LDH activity in C2BBe1 cells subsequent to chokeberry and blueberry pomace exposure (Figure 1d) is very suggestive of cell damage and is commonly linked with necrosis rather than apoptosis [79]. In contrast, TSLP is a cytokine known for its reported anti-tumor effects, directly promoting apoptosis in colon cancer cells [80]. The expression of TSLP was notably induced by all three pomace types, particularly by blueberry pomace, in both cell lysate and culture media (Table 6 and Table 7). This observation strongly suggests that apoptosis is induced in colorectal cancer enterocytes. TNFR1 is a membrane bound receptor of TNF-α, and mediates the induction of apoptosis. sTNFR1 is a soluble form of the receptor that acts as a decoy to regulate TNF-α activity by binding to it in the bloodstream. In human colorectal carcinoma tumors sTNFR1 was shown to be significantly upregulated compared to normal colon tissue [1], contributing to the insensitivity to apoptosis by TNF-α. Interestingly, under our experimental conditions, sTNFR1 was detected exclusively in the cell culture media, with levels significantly reduced compared to the control across all types of pomace treatment. This finding suggests that a 50 mg/mL dose of blueberry, chokeberry, and their mixture may enhance the sensitivity of tumor cells to pro-apoptotic stimuli such as TNF-α.

The Erk1/2 signaling cascades play pivotal roles in promoting cell proliferation and exerting anti-apoptotic effects by facilitating cell cycle progression, angiogenesis, and cellular survival [81]. Notably, despite its well-established anti-apoptotic role, accumulating evidence suggests that Erk1/2 may also possess pro-apoptotic functions [82]. This dual role could be explained by the potential inhibition of STAT1 signaling under conditions of elevated Erk1/2 activity [83], which in our case is in agreement with the results. In this study, we reported p-Erk1/2 expression was overall elevated compared to the control, particularly in the case of chokeberry pomace and pomace mix (Figure 1g,h). Conversely, STAT3 activation is typically associated with autocrine stimulatory loops in cancer, providing a growth advantage to the cells [84]. Activation of STAT3 in cancer cells is associated with resistance to apoptosis and increased malignancy, as it promotes tumor invasion and progression [85]. Thus, we suggest that inhibition of STAT3 protein expression following exposure to chokeberry and blueberry pomace support beneficial pro-apoptotic and anti-metastatic effects. The reduced level of gp130 we reported could explain both STATs inhibitions, as this cytokine was shown to be crucial for activating JAK/STAT molecules (JAK1, JAK2, Tyk2, STAT1, STAT3, STAT5) and the PI3K/AKT and MAPK [86]. We also reported a significant inhibition of Aky-1 under chokeberry pomace and the pomace mix, but not the blueberry pomace (Figure 2e,f). This result further strengthens the idea of cell death signaling pathways activation, as AKT deactivation characterizes both caspase-dependent and -independent cell death [87] and Akt-1 inhibition is considered a promising target for cancer therapy development [88]. Despite the limited number of studies conducted in this specific area, our findings appear to align with previous literature indicating anthocyanin-rich extracts from pigmented potato powder inhibit AKT-mTOR signalling in U-937 cells [89].

While the oxidized protein levels were not significantly altered by exposure to any pomace, MDA-protein adducts were particularly inhibited by both chokeberry and blueberry pomace treatment (Figure 2a–d). This finding is in agreement with previous literature reports indicating wild blueberry did not change protein carbonyls in TNF-α challenged and unchallenged Caco-2 cells [76], while polyphenol compounds from chokeberries were reported to significantly reduce lipid peroxidation [90].

The cytotoxic effects of chokeberry and blueberry pomace, as demonstrated by the significant reduction in cell viability observed in C2BBe1 cells following treatment, suggests a potent impact on inhibiting cancer cell growth and survival. In contrast, while the antioxidative properties are notable, as evidenced by the inhibition of MDA-protein adducts they may not be as central to their biological activity in this context compared to their cytotoxic effects. This distinction underscores the chokeberry and blueberry potential as effective agents in cancer therapy, primarily through their ability to target cancer cells directly rather than solely relying on antioxidative mechanisms.

## 5. Conclusions

Our study offers a comprehensive analysis of the anti-inflammatory and antitumoral properties of chokeberry and blueberry pomace aqueous extracts on C2BBe1 colorectal carcinoma cells. We developed a mathematical model to evaluate the antioxidative activity of these extracts, revealing that anthocyanins play a predominant role, with their effective contribution highlighted by a cubic function. This underscores the critical importance of anthocyanins among other antioxidant compounds. Using conventional extraction techniques, we found that chokeberry extracts consistently exhibited higher total polyphenol content, anthocyanin levels, and antioxidative activity compared to blueberry extracts.

**Figure 4:**
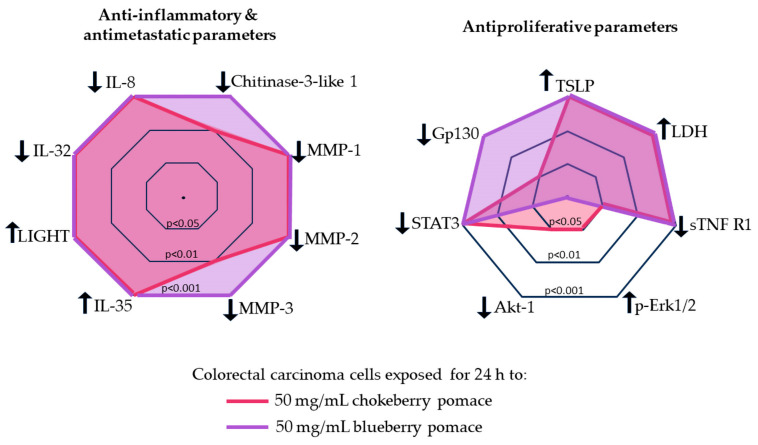
Comparison of the anti-inflammatory, anti-metastatic and anti-proliferative potential of the chokeberry and blueberry pomace extracts on C2BBe1 cells. Downward arrows indicate parameters expression decreased, while upward arrows indicate parameters expression increased. Concentric polygons represent the statistical significance of the parameter change vs. the control: inner polygon *p* < 0.05; middle polygon *p* < 0.01, outer polygon *p* < 0.001.

Despite the superior antioxidative profile of chokeberry, blueberry extracts had more pronounced antiproliferative and anti-inflammatory effects on C2BBe1 cells. Both chokeberry and blueberry pomace treatments led to non-inflammatory cell death characterized by loss of membrane integrity, reducing the risk of inflammatory responses—a desirable outcome in cancer therapy. While the cellular changes induced by both berry extracts generally followed the same direction, blueberry extract produced more consistent and significant alterations (Figure 4), suggesting distinct pathways of action for the two berry extracts. Our data indicated that the in vitro antioxidative effects of both pomaces were similar at a 50 mg/mL dose, thus the anti-inflammatory, anti-metastatic and antiproliferative effects may not be directly mediated by the antioxidative properties.

Chokeberry pomace extract’s effects appeared to be mediated through pathways involving Erk signaling and Akt-1 inhibition. Conversely, our results suggest that the inhibition gp130 and downregulation of STAT3 subsequent to the exposure to both pomace extracts, especially blueberry pomace, can enhance antiproliferative and antimetastatic effects in cancer cells. Advanced techniques such as transcriptomic and proteomic analyses should be used to further clarify the roles of these key regulatory molecules. Exploring the synergistic effects of these bioactive compounds with conventional cancer therapies could lead to improvements of treatment efficacy. Future research should also explore how blueberry and chokeberry pomace aqueous extract treatments affect additional cellular functions, such as cell adhesion and migration, in colorectal carcinoma cells. A comprehensive understanding of these molecular interactions will provide valuable insights for developing targeted nutraceuticals and functional foods for cancer prevention and therapy.

## Figures and Tables

**Table 1:** Tested hypotheses and model equations corresponding to these hypotheses, with validity conditions for the parameters of these equations.

Assumption	Statement of the Assumption	Equation
H1	The antioxidant activity (*y*) is determined only by the total concentration of polyphenols (*x*_1_)	$y=a\cdot x^{m}+c$ a,c>0
H2	The antioxidant activity (*y*) is predominantly determined by the concentration of anthocyanins (*x*_2_)	$y=b\cdot x^{n}+c$ b,c>0
H3	The antioxidant activity (*y*) is determined by the combined effects of polyphenols (*x*_1_) and anthocyanins (*x*_2_) in different proportions and according to different laws.	$y=a\cdot {\left(x_{1}-x_{2}\right)}^{m}+b\cdot x_{2}^{n}+c$ a,b,c>0

**Table 2:** The TPC in mg of gallic acid equivalents per gram of dry weight (mg GAE/g dw) pomace in different extraction solvents.

		Extraction Conditions				
		50% Ethanol	70% Ethanol	100% Ethanol	0.5% Vinegar	Distilled **Water**
Chokeberry (Cb)	Mean (mg GAE/g dw) ± SD	48.6 ± 1.3	47.3 ± 1.1	51.1 ± 1.5	17.7 ± 0.3	9.0 ± 1.0
	50% Ethanol				*p* < 0.001	*p* < 0.001
	70% Ethanol			*p* < 0.05	*p* < 0.001	*p* < 0.001
	100% Ethanol				*p* < 0.001	*p* < 0.001
	0.5% Vinegar					*p* < 0.001
Blueberry (Bb)	Mean (mg GAE/g dw) ± SD	10.8 ± 0.2	11.2 ± 0.5	4.1 ± 0.1	4.1 ± 0.2	3.8 ± 0.4
	50% Ethanol			*p* < 0.001	*p* < 0.001	*p* < 0.001
	70% Ethanol			*p* < 0.001	*p* < 0.001	*p* < 0.001
	100% Ethanol					
	0.5% Vinegar					
*p*-value Cb vs. Bb		*p* < 0.001	*p* < 0.001	*p* < 0.001	*p* < 0.001	*p* < 0.01

**Table 3:** The total anthocyanins content and composition (%) of pomaces in different extraction solvents.

			Extraction Conditions		
			50% Ethanol	70% Ethanol	0.5% Vinegar
Chokeberry (Cb)	Total anthocyanins (mg/g dw) Mean ± SD		3.05 ± 0.49	2.26 ± 0.36	1.58 ± 0.17
	Anthocyanins composition (%)	Cyn *3-O*-Gal	68.9	64.6	75.0
		Cyn *3-O*-Glu	1.6	2.0	1.7
		Cyn *3-O*-Arb	20.0	21.0	16.8
		Cyn *3-O*-Xyl	9.5	12.3	6.4
	*p*-value	50% Ethanol			*p* < 0.01
		70% Ethanol			*p* < 0.05
Blueberry (Bb)	Total anthocyanins (mg/g dw) Mean ± SD		3.15 ± 0.07	2.91 ± 0.03	0.83 ± 0.02
	Anthocyanins composition (%)	Del *3-O*-Gal	18.5	17.3	nd
		Cyn *3-O*-Gal	13.1	13.6	21.7
		Cyn *3-O*-Glu	7.3	6.2	nd
		Others	61.1	62.9	78.8
	*p*-value	50% Ethanol		*p* < 0.01	*p* < 0.001
		70% Ethanol			*p* < 0.001
*p*-value Total anthocyanins Cb vs. Bb				*p* < 0.05	*p* < 0.001

**Table 4:** Antioxidant activities of chokeberry and blueberry pomace extracts in different solvents.

		Extraction Conditions				
		50% Ethanol	70% Ethanol	100% Ethanol	0.5% Vinegar	Distilled Water
Chokeberry (Cb)	Mean (mg TE/g dw) ± SD	684 ± 18	697 ± 10	610 ± 7	298 ± 28	216 ± 8
	50% Ethanol			*p* < 0.01	*p* < 0.001	*p* < 0.001
	70% Ethanol			*p* < 0.001	*p* < 0.001	*p* < 0.001
	100% Ethanol				*p* < 0.001	*p* < 0.001
	0.5% Vinegar					*p* < 0.01
Blueberry (Bb)	Mean (mg TE/g dw) ± SD	120 ± 2	127 ± 2	62 ± 2	50 ± 3	53 ± 1
	50% Ethanol		*p* < 0.01	*p* < 0.001	*p* < 0.001	*p* < 0.001
	70% Ethanol			*p* < 0.001	*p* < 0.001	*p* < 0.001
	100% Ethanol				*p* < 0.01	*p* < 0.001
	0.5% Vinegar					
*p*-value Cb vs. Bb		*p* < 0.001	*p* < 0.001	*p* < 0.001	*p* < 0.001	*p* < 0.001

**Table 5:** Coefficients of the models for antioxidant activity according to the concentrations of polyphenols and anthocyanins obtained by extractions in aqueous and ethanolic solutions. N/A—not applicable.

Assumption on the Determinant of Antioxidant Activity (y)	Model Label	Model Name	Equation	m	n	a	b	c	ER
H1 Polyphenols $\left(x_{1}\right)$	H1.1	Linear	$y=ax_{1}+c$	1	N/A	7.50	N/A	43.66	133.00
	H1.2	Quadratic	$y=ax_{1}^{2}+c$	2	N/A	0.13	N/A	94.83	134.45
	H1.3	Cubic	$y=ax_{1}^{3}+c$	3	N/A	<0.01	N/A	104.86	135.59
	H1.4	Fractional power	$y=ax_{1}^{m}+c$	0.93	N/A	9.89	N/A	35.24	132.99
H2 Anthocyanins $(x_{2}$)	H2.1	Linear	$y=bx_{2}+c$	N/A	1	N/A	−32.51	189.41	64.04
	H2.2	Quadratic	$y=bx_{2}^{2}+c$	N/A	2	N/A	0	118.31	72.55
	H2.3	Cubic	$y=bx_{2}^{3}+c$	N/A	3	N/A	1.66	1.02	67.42
	H2.4	Fractional power	$y=bx_{2}^{m}+c$	N/A	0.24	N/A	54.33	0.00	59.07
H3 Polyphenols without anthocyanins (*x*_1_−*x*_2_) and anthocyanins (*x*_2_)	H3.1	Linear	$y=a\left(x_{1}-x_{2}\right)+bx_{2}+c$	1	1	0.00	23.74	22.95	57.90
	H3.2	Quadratic	$y=a\left(x_{1}-x_{2}\right)+bx_{2}^{2}+c$	1	2	0.00	7.79	29.05	56.17
	H3.3	Cubic	$y=a\left(x_{1}-x_{2}\right)+bx_{2}^{3}+c$	1	3	1.01	1.64	1.02	51.76
	H3.4	Partial fractional	$y=a\left(x_{1}-x_{2}\right)+bx_{2}^{n}+c$	1	4.95	0.00	0.26	39.44	55.00
	H3.5	Fractional	$y=a{\left(x_{1}-x_{2}\right)}^{m}+bx_{2}^{n}+c$	2.75	6.36	0.03	0.08	0.00	55.55

**Table 6:** Cytokine levels in whole cell lysate.

Lysate	Control	Chokeberry (Cb) 50 mg/mL		Blueberry (Bb) 50 mg/mL		Cb & Bb 50 mg/mL	
Cytokine	Average (pg/mL)	Average (pg/mL)	Fold change *p*-value	Average (pg/mL)	Fold change *p*-value	Average (pg/mL)	Fold change *p*-value
Chitinase-3-like 1	143.9 ± 4.7	49.8 ± 1.4	0.4 *p* < 0.001	35.5 ± 2.2	0.3 *p* < 0.001	42.7 + 0.9	0.3 *p* < 0.001
gp130	180.0 ± 5.8	165.0 ± 3.3	0.9 *p* < 0.05	110.9 ± 1.3	0.6 *p* < 0.001	115.3 ± 1.0	0.6 *p* < 0.001
IL-8	42.3 ± 2.5	24.2 ± 0.7	0.6 *p* < 0.001	14.4 ± 0.7	0.4 *p* < 0.001	21.7 ± 1.0	0.5 *p* < 0.001
IL-32	62.1 ± 0.9	36.8 ± 1.5	0.6 *p* < 0.001	26.8 ± 2.3	0.4 *p* < 0.001	33.3 ± 1.9	0.5 *p* < 0.001
IL-35	21.1 ± 0.6	43.9 ± 0.3	2.1 *p* < 0.001	57.1 ± 0.5	2.7 *p* < 0.001	53.3 ± 0.6	2.5 *p* < 0.001
LIGHT	14.0 ± 0.4	35.5 ± 0.8	2.4 *p* < 0.001	45.4 ± 0.3	3.2 *p* < 0.001	36.1 ± 0.7	2.6 *p* < 0.001
MMP-1	87.1 ± 0.6	54.4 ± 0.7	0.6 *p* < 0.001	42.4 ± 0.7	0.5 *p* < 0.001	49.6 ± 0.4	0.6 *p* < 0.001
MMP-2	104.8 ± 1.3	72.9 ± 1.1	0.7 *p* < 0.001	57.9 ± 1.1	0.6 *p* < 0.001	63.2 ± 0.7	0.6 *p* < 0.001
MMP-3	36.4 ± 0.4	29.1 ± 0.8	0.8 *p* < 0.001	21.1 ± 0.1	0.6 *p* < 0.001	27.8 ± 0.2	0.8 *p* < 0.001
TSLP	18.8 ± 0.5	43.4 ± 0.4	2.3 *p* < 0.001	89.3 ± 0.5	4.8 *p* < 0.001	72.4 ± 0.5	3.9 *p* < 0.001

**Table 7:** Cytokine levels in cell culture media.

Culture Medium	Control	Chokeberry (Cb) 50 mg/mL		Blueberry (Bb) 50 mg/mL		Cb& Bb 50 mg/mL	
Cytokine	Average (pg/mL)	Average (pg/mL)	Fold change *p*-value	Average (pg/mL)	Fold change *p*-value	Average (pg/mL)	Fold change *p*-value
Chitinase-3-like 1	286.2 ± 15.8	76.1 ± 39.6	0.3 *p* < 0.01	78.2 ± 1.5	0.3 *p* < 0.001	85.4 ± 0.8	0.3 *p* < 0.001
gp130	257.2 ± 24.4	103.7 ± 6.1	0.4 *p* < 0.001	64.7 ± 3.1	0.3 *p* < 0.001	89.7 ± 1.6	0.4 *p* < 0.001
IL-8	64.1 ± 1.3	29.8 ± 1.0	0.5 *p* < 0.001	19.1 ± 0.5	0.3 *p* < 0.001	23.4 ± 0.5	0.4 *p* < 0.001
IL-32	76.7 ± 0.3	58.9 ± 0.8	0.8 *p* < 0.001	50.4 ± 0.7	0.7 *p* < 0.001	52.4 ± 1.1	0.7 *p* < 0.001
IL-35	26.9 ± 0.2	61.4 ± 1.2	2.3 *p* < 0.001	97.6 ± 4.2	3.6 *p* < 0.001	68.6 ± 1.4	2.6 *p* < 0.001
LIGHT	17.7 ± 0.2	55.0 ± 0.6	3.1 *p* < 0.001	74.4 ± 1.1	4.2 *p* < 0.001	65.5 ± 0.6	3.7 *p* < 0.001
MMP-1	138.6 ± 0.6	65.9 ± 0.5	0.5 *p* < 0.001	47.8 ± 0.7	0.3 *p* < 0.001	60.7 ± 0.5	0.4 *p* < 0.001
MMP-2	167.7 ± 0.4	84.9 ± 0.5	0.5 *p* < 0.001	61.6 ± 0.6	0.4 *p* < 0.001	74.0 ± 0.3	0.4 *p* < 0.001
MMP-3	43.3 ± 0.9	35.3 ± 0.9	0.8 *p* < 0.01	27.9 ± 0.5	0.6 *p* < 0.001	31.6 ± 0.6	0.7 *p* < 0.001
sTNF R1	41.0 ± 2.5	16.6 ± 4.2	0.4 *p* < 0.001	13.2 ± 0.7	0.3 *p* < 0.001	13.8 ± 0.4	0.3 *p* < 0.001
TSLP	31.4 ± 0.3	187.8 ± 1.5	6.0 *p* < 0.001	288.5 ± 2.6	9.2 *p* < 0.001	241.3 ± 1.0	7.7 *p* < 0.001

## Data Availability

Data supporting this study are included within the article and Supporting Materials, further inquiries can be directed to the corresponding author.

## Supplementary Materials

The following supporting information can be downloaded at: https://www.mdpi.com/article/10.3390/foods13162552/s1, Figure S1: Chromatographic profile of anthocyanins at 520 nm of aqueous and ethanolic chokeberry extracts; Figure S2: Chromatographic profile of anthocyanins at 520 nm of aqueous and ethanolic blueberry extracts; Figure S3: Original images used for western blot analysis.
