# Supplementary material for: Development of Functional Foods: A Comparative Study on the Polyphenols and Anthocyanins Content in Chokeberry and Blueberry Pomace Extracts and Their Antitumor Properties
Source: Foods. 2024 Aug 16;13(16):2552. doi: 10.3390/foods13162552 (PMC11353723; doi:10.3390/foods13162552)
Supplement: Supplementary file 1 [file foods-13-02552-s001.zip › foods-3131201-supplementary.pdf]

Development of Functional Foods: A Comparative Study on the Polyphenols and Anthocyanins Content in Chokeberry and Blueberry Pomace Extracts and Their Antitumor and Antioxidative Properties

Loredana Stanca[1,†], Liviu Bilteanu [1,†], Oana Crina Bujor [2], Violeta Alexandra Ion [2], Andrei Cătălin Petre [2], Liliana Bădulescu [2], Ovidiu Ionut Geicu [1], Aurelia Magdalena Pisoschi [1], Andreea Iren Serban [1,3*], Oana-Mărgărita Ghimpeteanu [1]

[1] Faculty of Veterinary Medicine, University of Agronomic Sciences and Veterinary Medicine Bucharest, 105 Blvd, Splaiul Independenței, 050097 Bucharest, Romania; loredana.stanca@fmvb.usamvb.ro; liviu.bilteanu@fmvb.usamvb.ro; ovidiu-ionut.geicu@fmvb.usamvb.ro; aurelia-magdalena.pisoschi@fmvb.usamv.ro; margarita.ghimpeteanu@fmvb.usamv.ro;

[2] Research Center for Studies of Food Quality and Agricultural Products, University of Agronomic Sciences and Veterinary Medicine of Bucharest, 59 Mărăști Blvb, 011464 Bucharest, Romania; oana.bujor@qlab.usamv.ro; violeta.ion@qlab.usamv.ro; andrei.petre@qlab.usamv.ro; liliana.badulescu@usamv.ro;

[3] Faculty of Biology, University of Bucharest, 91-95 Blvd, Splaiul Independenței, 050095 Bucharest, Romania;

* Andreea Iren Serban: andreea-iren.serban@fmvb.usamv.ro;

† These authors contributed equally to this work.

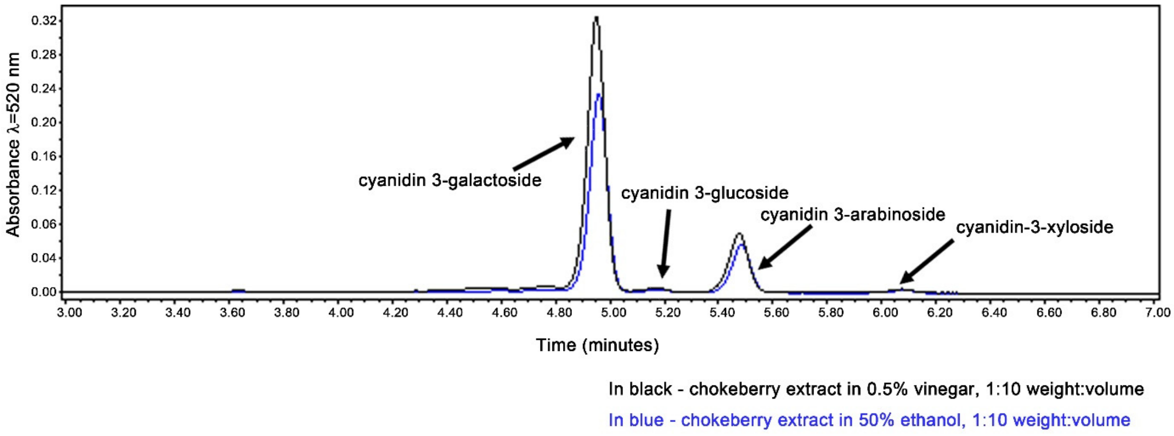

Figure S1. Chromatographic profile of anthocyanins at 520 nm of aqueous and ethanolic chokeberry extracts.

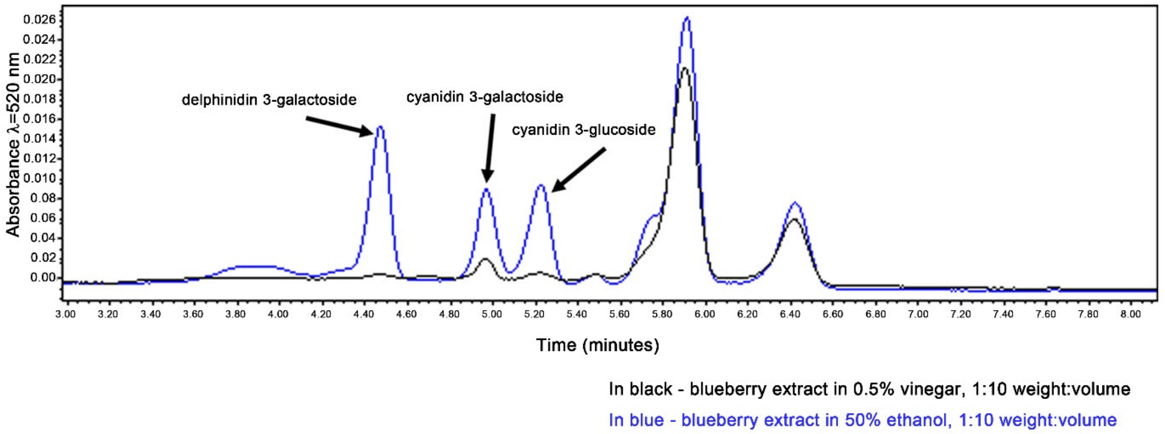

Figure S2. Chromatographic profile of anthocyanins at 520 nm of aqueous and ethanolic blueberry extracts.

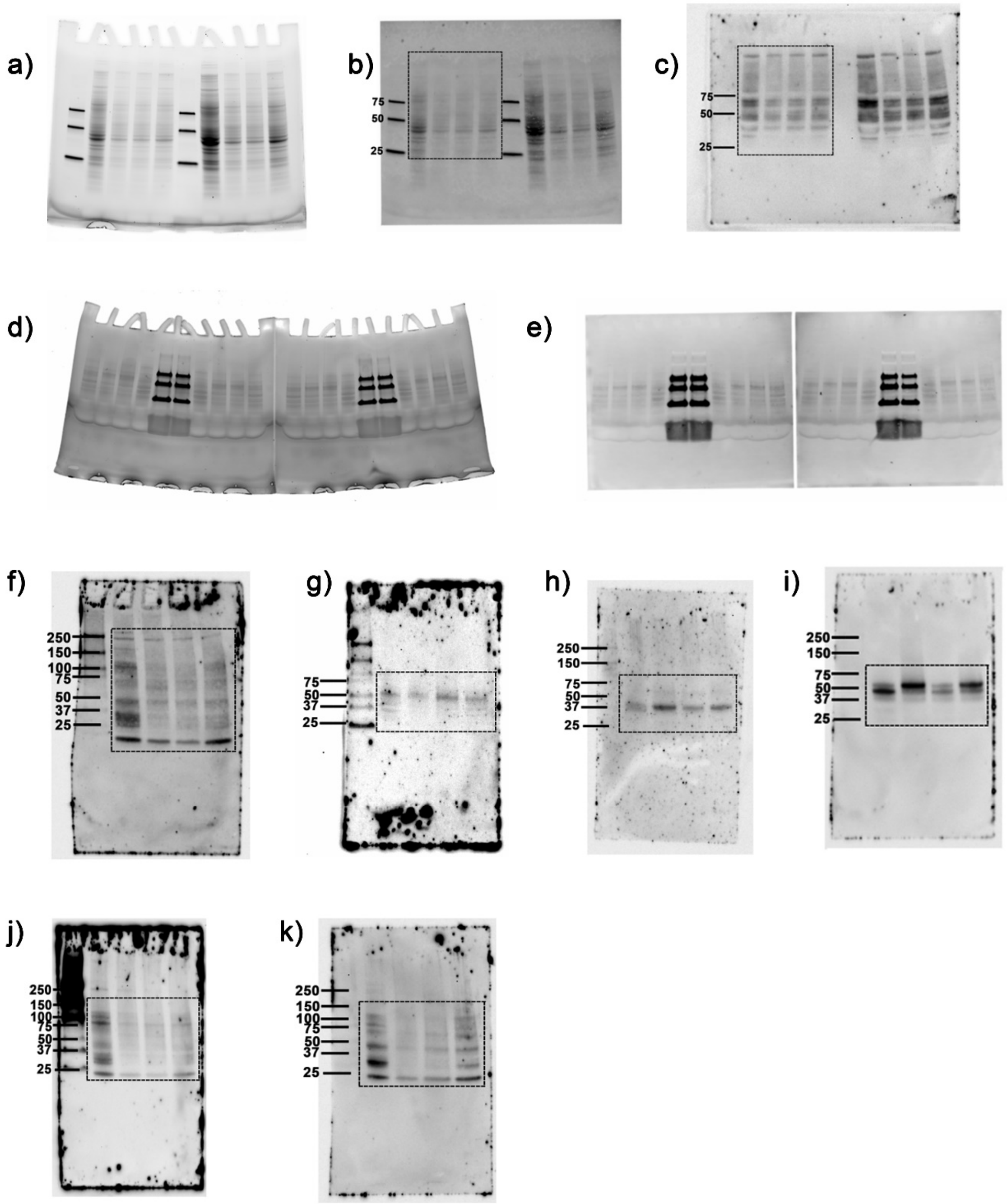

Figure S3. Original images used for western blot analysis. a) example of resolved SDS-PAGE gel used further for oxidized protein quantification: control is always to the right of the molecular ladder. Sample order: Control, chokeberry pomace treatment, blueberry pomace treatment, chokeberry and blueberry pomace mix treatment; b) The transferred membrane corresponding to a), with the area used for total protein normalization indicated in a dotted rectangle; c) Oxidized proteins blotted membrane with indication of the section shown in the paper; d) Example of resolved SDS PAGE gels before electrotranfer,

which were used for MDA-protein adducts, Akt-1, p-Erk1/2, STATS and occludin protein quantification, with controls always near the MW marker, samples loaded in mirror relative to the center. Sample order, from left to right chokeberry and blueberry pomace mix treatment, blueberry treatment, chokeberry pomace treatment, control, molecular ladder, molecular ladder, control, chokeberry pomace treatment, blueberry pomace treatment, chokeberry and blueberry pomace mix treatment. e) example of transferred membranes used for MDA-protein adducts, Akt-1, p-Erk1/2, STATS and occluding. The same loading order as indicated in d). For specific antibody incubation, the membrane was cut along the center, between the molecular ladders, after the membrane blocking step. For the blot images which were loaded in reverse order, the horizontally flipped version is presented. f) MDA-protein adducts blotted membrane; g) Akt-1 blotted membrane; h) p-Erk1/2 blotted membrane; i) occludin blotted membrane; j) STAT1 blotted membrane; k) STAT3 blotted membrane. In f-k, the dotted rectangles indicate the part which was cropped and presented in Figure 2.
